# A DNA Tetrahedron Delivery Asiatic Acid to Reprogram Mitochondrial Metabolism for Promoting Bone Regeneration via STAT3 Phosphorylation

**DOI:** 10.1002/advs.202518796

**Published:** 2025-12-19

**Authors:** Yiwen Huang, Yiming Zhang, Yisheng Feng, Qixiang Yang, Beiyuan Gao, Haiyang Xu, Cheng Huang, Kaili Lin, Yuanzhi Xu, Peiqi Zhu

**Affiliations:** ^1^ Department of Stomatology Shanghai Tenth People's Hospital School of medicine Tongji University Shanghai 200092 P. R. China; ^2^ Department of Oral and Cranio‐Maxillofacial Surgery Shanghai Ninth People's Hospital Shanghai Jiao Tong University School of Medicine College of Stomatology Shanghai Jiao Tong University Shanghai 200011 P. R. China; ^3^ National Center for Stomatology National Clinical Research Center for Oral Diseases Shanghai Key Laboratory of Stomatology Shanghai Research Institute of Stomatology Shanghai 200011 P. R. China; ^4^ Periodontology Unit Centre for Host‐Microbiome Interactions Faculty of Dentistry Oral and Craniofacial Sciences King's College London London SE1 9RT UK; ^5^ Department of Stomatology Shanghai Fourth People's Hospital School of Medicine Tongji University Shanghai 200011 P. R. China

**Keywords:** angiogenesis, bone regeneration, DNA tetrahedron nanostructures, metabolic reprogramming, mitochondrial targeting

## Abstract

Craniofacial bone defects remain a significant clinical challenge due to the complex healing process among immune regulation, vascularization, and osteogenesis. Asiatic acid (AA), a natural pentacyclic triterpenoid, has shown promise in modulating inflammation and promoting bone repair, yet its clinical application is hampered by poor solubility, low bioavailability, and lack of targeted delivery. Here, a multifunctional hydrogel‐integrated DNA nanostructure system is reported, in which AA‐loaded DNA tetrahedra are embedded within a Hyaluronic Acid Methacrylate (HAMA) hydrogel（HM‐TDN@AA） to enhance local retention, bioavailability, and controlled release. The HM‐TDN@AA system significantly inhibited osteoclastogenesis and enhanced the osteogenic and angiogenic activity of mesenchymal stem cells and endothelial cells, respectively. In vivo implantation in a calvarial defect model revealed early enhancement of vascularization and remodeling of the immune niche, followed by robust bone formation. Transcriptomic profiling of bone tissue uncovered a metabolic reprogramming signature characterized by activation of mitochondrial oxidative phosphorylation (OXPHOS) pathways. Network pharmacology and molecular docking further identified STAT3 as a key regulatory node targeted by AA. Collectively, the findings demonstrate that the HM‐TDN@AA platform orchestrates bone regeneration by simultaneously modulating inflammation, angiogenesis, and cellular metabolism. This study provides a novel strategy that integrates nanostructure‐assisted drug delivery with metabolic control to enhance osteoimmune coupling and vascularized bone regeneration.

## Graphical abstract

A hyaluronic acid methacrylate (HAMA) hydrogel incorporating DNA tetrahedrons loaded with Asiatic acid (TDN@AA) was developed. HM‐TDN@AA promotes angiogenesis of endothelial cells (ECs), inhibits osteoclastogenesis from bone marrow–derived macrophages (BMDMs), and enhances osteogenesis of mesenchymal stem cells (MSCs) via STAT3‐mediated mitochondrial metabolic reprogramming.

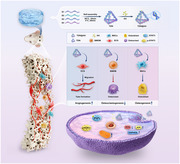

## 1 Introduction

Large craniofacial bone defects caused by trauma, tumor resection, or congenital anomalies remain a formidable clinical challenge due to the region's complex anatomical structure, limited vascular supply, and inherently poor regenerative capacity.^[^ ^1^ ^]^ While autologous and allogeneic bone grafts are widely used, they are often constrained by donor site morbidity, immune rejection, and poor long‐term integration.^[^ ^2^, ^3^ ^]^ Synthetic biomaterials have also been explored extensively, yet most lack bioactivity and fail to adequately modulate the local microenvironment.^[^ ^4^ ^]^ Successful bone regeneration, particularly in the craniofacial region, relies not only on osteo‐conductivity but also on the dynamic orchestration of immune modulation, angiogenesis, and osteogenesis.^[^ ^5^, ^6^, ^7^ ^]^ These biological events are tightly interconnected: macrophages initiate and resolve inflammation, endothelial cells form new vasculature and provide metabolic support, and osteoblasts reconstruct the bone matrix.^[^ ^8^, ^9^ ^]^ However, most existing biomaterials function as passive scaffolds and lack the capacity to coordinate this immune‐vascular‐osteogenic axis, resulting in suboptimal healing outcomes.^[^ ^10^, ^11^ ^]^ Therefore, there is an urgent need for multifunctional biomaterials capable of actively engaging with the regenerative microenvironment to promote robust and integrated bone repair.

Natural small molecules have drawn increasing attention in regenerative medicine due to their intrinsic bioactivity, low immunogenicity, and favorable safety profiles.^[^ ^12^, ^13^ ^]^ Asiatic acid (AA), a pentacyclic triterpenoid compound extracted from Centella asiatica, has demonstrated a wide range of therapeutic effects, including anti‐inflammatory, antioxidant, pro‐angiogenic, and osteo‐inductive properties.^[^ ^14^ ^]^ Such multifaceted regulatory capacity makes AA an attractive candidate for modulating the immune‐vascular‐osteogenic axis during bone regeneration. However, the translational potential of AA remains limited by several pharmacokinetic drawbacks. Its poor water solubility, rapid systemic clearance, and low bioavailability restrict its effective accumulation at bone defect sites.^[^ ^15^ ^]^ Additionally, inadequate cellular uptake or low delivery efficiency further limits its ability to exert sustained and localized effects within complex regenerative microenvironments.^[^ ^16^ ^]^ These limitations have hindered the clinical adoption of AA despite its promising biological profile, underscoring the need for an efficient delivery strategy to fully harness its therapeutic potential.

To address these challenges, advanced drug delivery platforms have been explored to enhance the stability, bioavailability, and spatial targeting of small molecules such as AA. Among various nanocarriers, DNA nanostructures, particularly DNA tetrahedrons (TDNs), have emerged as a promising class of programmable and biocompatible delivery platforms.^[^ ^17^ ^]^ TDNs are constructed through the self‐assembly of short synthetic oligonucleotides into well‐defined three‐dimensional architectures characterized by high structural uniformity, mechanical rigidity, and nanoscale precision.^[^ ^18^ ^]^ These features confer enhanced cellular uptake, low immunogenicity, and excellent stability, making TDNs suitable for biomedical applications such as targeted drug delivery, gene therapy, and tissue engineering.^[^ ^18^, ^19^ ^]^ Their intrinsic properties, such as enzymatic stability, low immunogenicity, and efficient cellular uptake via receptor‐mediated endocytosis, make them attractive vehicles for intracellular delivery.^[^ ^20^, ^21^ ^]^ Moreover, TDNs possess excellent structural tunability and can be engineered to encapsulate and deliver small‐molecule drugs, nucleic acids, and proteins, providing a versatile platform for a wide range of biomedical applications.^[^ ^22^, ^23^ ^]^ To further improve local retention and sustained release, TDNs can be incorporated into injectable hydrogels such as Hyaluronic Acid Methacrylate (HAMA) to create a hybrid material system. HAMA hydrogels provide a hydrated and supportive microenvironment, enable minimally invasive delivery, and offer spatial confinement of therapeutic agents.^[^ ^24^, ^25^, ^26^ ^]^ By embedding TDNs within the HAMA matrix, it is possible to achieve prolonged release kinetics, enhanced structural stability, and improved cellular accessibility.^[^ ^27^, ^28^ ^]^ This combinatorial strategy holds great potential for delivering therapeutic agents in a spatiotemporally controlled manner to modulate the immune‐vascular‐osteogenic interface during bone regeneration.

**Scheme 1:**
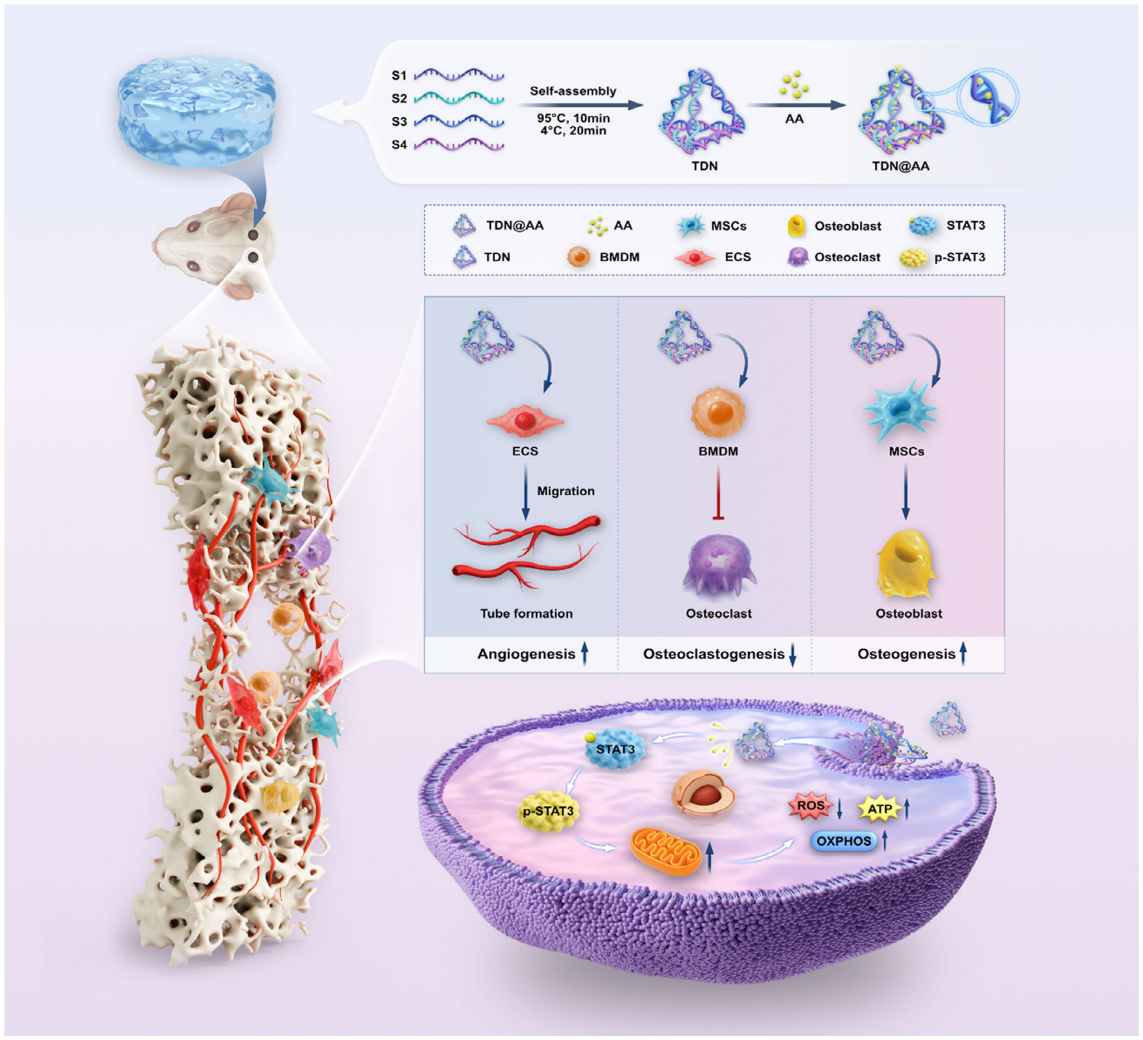
A metabolically active hydrogel (HM‐TDN@AA) was developed by embedding asiatic acid (AA) loaded DNA tetrahedrons (TDN@AA) into a methacrylated hyaluronic acid (HAMA) matrix. While the hydrogel itself is biologically inert, it enables metabolically active modulation of local cells by sustainably delivering AA. Mechanistically, HM‐TDN@AA activates STAT3 phosphorylation and enhances mitochondrial oxidative phosphorylation (OXPHOS), resulting in increased ATP production and reduced ROS levels. These metabolic effects promote angiogenesis, suppress osteoclastogenesis, and enhance osteogenesis, thereby establishing a regenerative immune–vascular–osteogenic microenvironment conducive to bone repair.

In this study, we developed a HAMA‐supported TDN platform (HM‐TDN@AA) to achieve stabilized and enhanced delivery of Asiatic acid for bone regeneration. We hypothesized that this delivery system could overcome the pharmacokinetic limitations of AA and simultaneously coordinate immune modulation, angiogenesis, and osteogenesis through STAT3 and activation of mitochondrial reprogramming. A combination of in vitro and in vivo assays, including cell‐based functional evaluations, transcriptomic profiling, and bone defect modeling, was employed to validate this strategy. Our findings provide a mechanistically grounded framework for integrating natural small molecules with gene‐programmable nanostructures, offering a novel approach to promote immuno‐vascular coupled bone regeneration (**Scheme** **1**).

## 2 Results and Discussion

### 2.1 Construction and Characterization of TDN@AA Nanostructures

**Figure 1:**
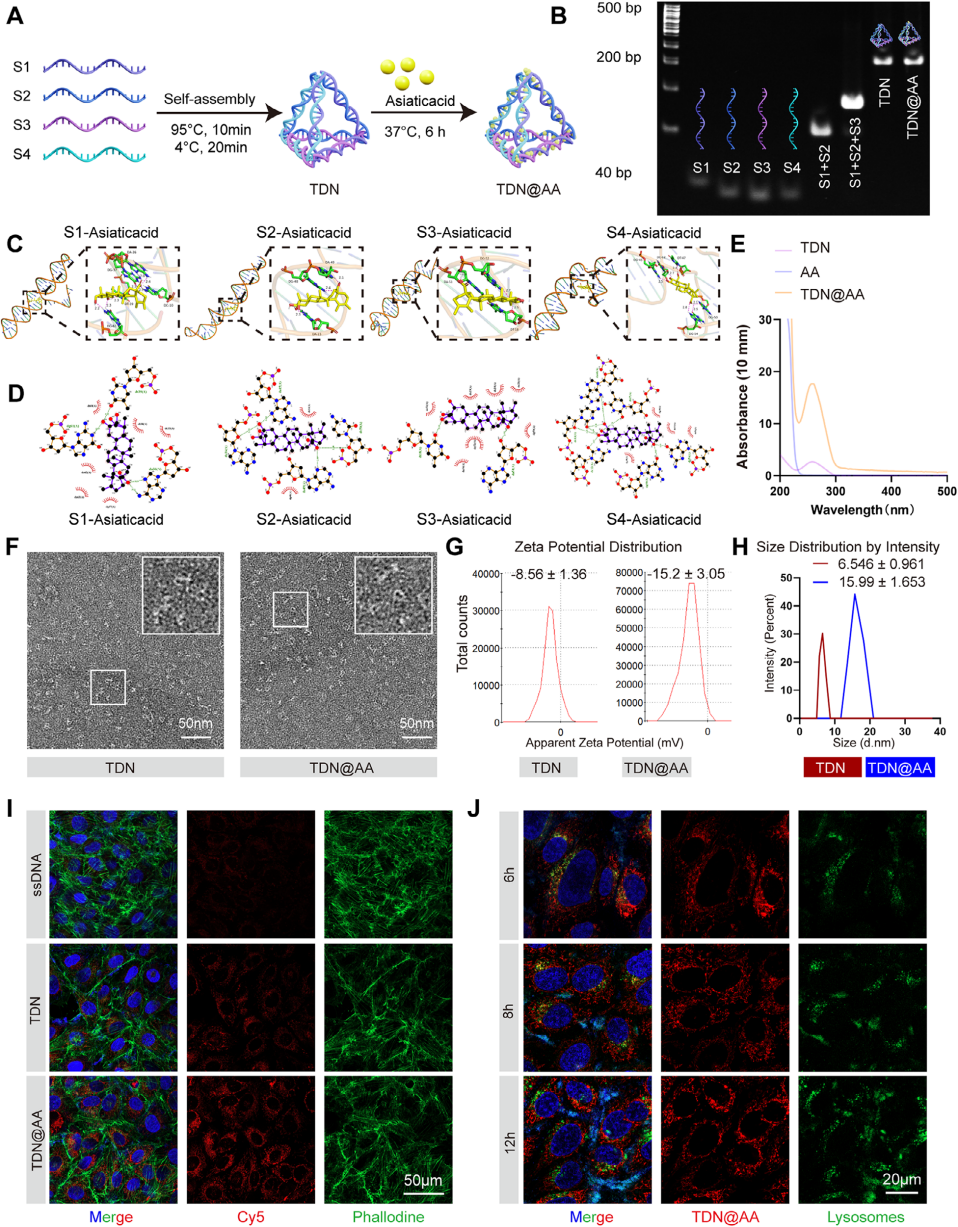
Design and characterization of TDN@AA nanostructures. A) Schematic representation of the self‐assembly of four single‐stranded DNAs (S1‐S4) into a DNA tetrahedron (TDN), followed by loading of Asiatic acid (AA). B) Agarose gel electrophoresis confirming the successful assembly of TDN and formation of TDN@AA. C) Molecular docking of AA with individual DNA strands (S1‐S4), showing predicted binding interactions within the DNA backbone. D) Structural modeling of AA interactions with DNA strands, highlighting hydrogen bonds and non‐covalent interactions. E) UV–Vis absorption spectra of free AA, TDN, and TDN@AA, demonstrating characteristic peak shifts upon drug loading. F) TEM images reveal a triangular‐shaped structure of both TDN and TDN@AA. Scale bar: 50 nm. G) Zeta potential measurements showing surface charge changes before and after AA loading (*n* = 3/group). H) Dynamic light scattering analysis of particle size distribution, revealing an increase in hydrodynamic diameter for TDN@AA compared to TDN alone (*n* = 3/group). I) Immunofluorescence images demonstrate cellular uptake of Cy5‐labeled ssDNA, TDN, and TDN@AA by HUVECs (Cy5: red; cytoskeleton: green; nucleus: blue) (Scale bar: 50 µm). J) Time‐dependent lysosomal escape of TDN@AA at 6, 8, and 12 h. Lysosomes: LysoTracker (green); TDN@AA: Cy5 (red); nuclei: DAPI (blue). Scale bar: 10 µm. (*n* = 3/group).

To improve the bioavailability and targeted delivery of Asiatic acid (AA), we designed a programmable DNA tetrahedron nanostructure (TDN) for AA encapsulation. As illustrated in **Figure** **1**A, the TDNs were assembled by mixing four single‐stranded oligonucleotides (S1‐S4)^[^ ^29^ ^]^ via a thermal annealing strategy (Table S1, Supporting Information). Subsequent incubation with AA at 37 °C allowed efficient loading through hydrophobic interactions and potential hydrogen bonding with nucleotide bases.^[^ ^30^ ^]^ Successful self‐assembly was first confirmed by gel electrophoresis (Figure 1B). The four individual strands (S1‐S4) migrated at expected sizes, while the assembled TDN showed a significantly slower migration pattern, indicative of successful higher‐order structure formation. Notably, the TDN@AA complex displayed a similar migration profile to that of pure TDN, suggesting that AA loading did not disrupt the integrity of the tetrahedral scaffold.

To further elucidate the binding conformation of AA with DNA strands, molecular docking was performed on all four single strands. As shown in Figure 1C,D, AA established stable non‐covalent interactions, primarily through hydrogen bonding and van der Waals forces, with the nucleobases in each strand, indicating its capacity for multivalent binding.^[^ ^31^ ^]^ These molecular simulations support the hypothesis that AA is stably accommodated within or around the DNA scaffold without covalent modification. UV–Vis spectroscopy further verified AA incorporation into TDNs. As shown in Figure 1E, TDN@AA exhibited characteristic absorbance peaks overlapping both free TDN and AA, confirming successful drug loading.

Transmission electron microscopy (TEM) revealed uniformly dispersed tetrahedral nanostructures with sharp edges and consistent morphology (Figure 1F). Importantly, no aggregation was observed after AA loading, suggesting preserved colloidal stability. Zeta potential analysis showed that both TDN and TDN@AA maintained negative surface charges (Figure 1G), with a slight decrease in absolute potential after AA loading (from −8.56 to −15.2 mV), likely due to neutralization of negative DNA phosphate groups by hydrophobic AA.^[^ ^29^, ^30^ ^]^ Dynamic light scattering (DLS) confirmed an increase in hydrodynamic diameter from 6.5 nm (TDN) to 16.0 nm (TDN@AA), consistent with successful loading of AA molecules (Figure 1H). To investigate cellular uptake, Cy5‐labeled TDN and TDN@AA were incubated with HUVECs. Confocal microscopy showed robust cytoplasmic accumulation of both nanostructures, while negligible signal was detected for free ssDNA, indicating the necessity of the tetrahedral architecture for effective internalization (Figure 1I). Furthermore, lysosomal colocalization analysis at 6, 8, and 12 h post‐incubation revealed that TDN@AA initially accumulated in lysosomes but gradually relocated into the cytoplasm (Figure 1J), confirming its effective lysosomal escape that serves as a prerequisite for intracellular bioactivity.

Together, these data demonstrate that AA can be efficiently loaded onto DNA tetrahedrons via non‐covalent interactions without compromising structural integrity, colloidal stability, or particle size uniformity. This nanoscale platform offers a rationally engineered vehicle for the controlled delivery of AA in tissue regeneration applications.

### 2.2 Preparation and Characterization of HM‐TDN@AA Loaded HAMA Hydrogel

**Figure 2:**
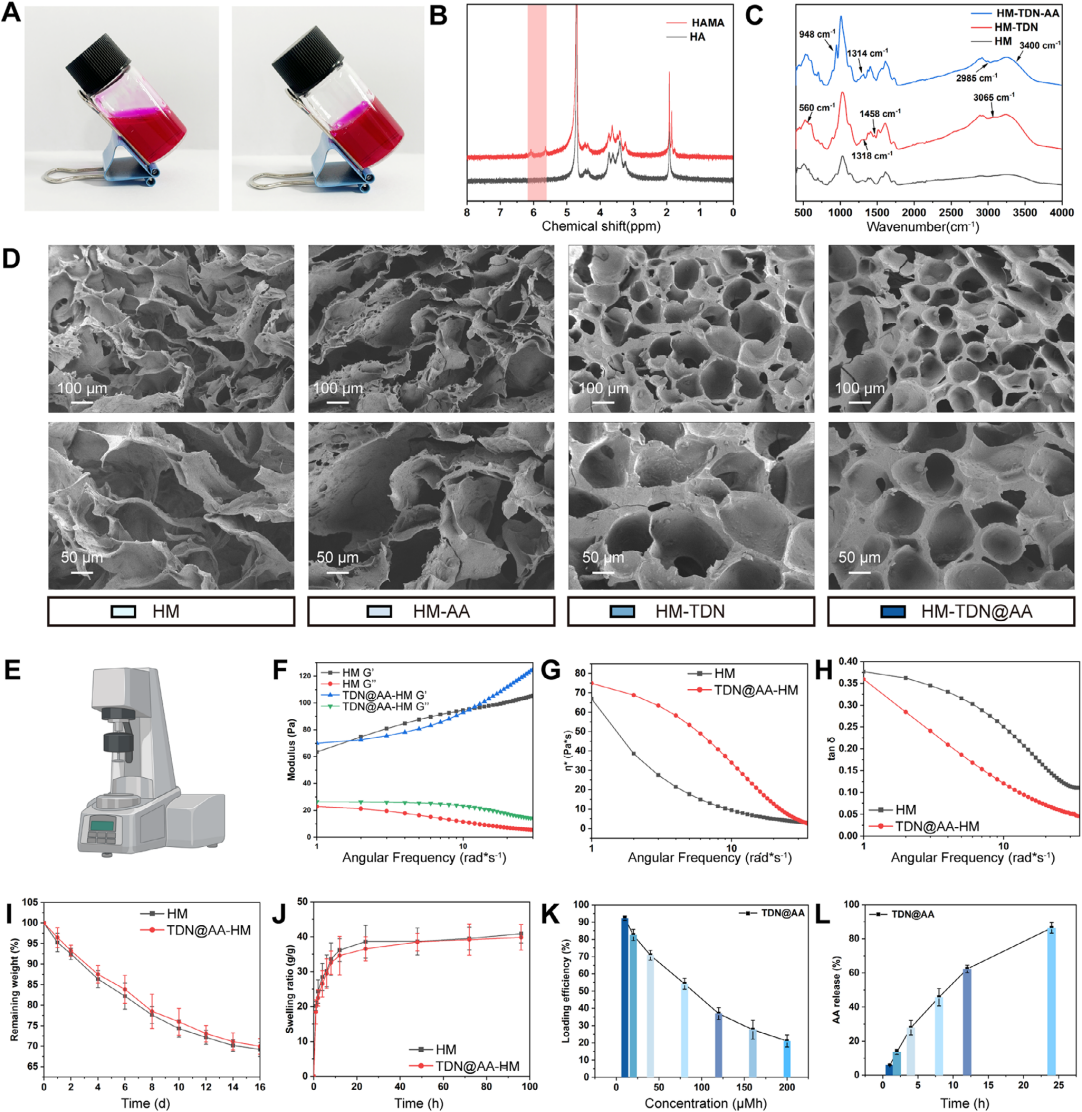
Physicochemical characterization of HM‐TDN@AA‐loaded hydrogel. A) Photographs showing the injectable precursor solution before and after UV‐induced hyaluronic acid. B) ^1^H NMR spectra of HA and HAMA, confirming successful methacrylation. C) FTIR spectra of HM, HM‐TDN, and HM‐TDN@AA indicating characteristic chemical bond shifts. D) SEM images showing internal pore structures of HM, HM‐AA, HM‐TDN, HM‐TDN@AA groups. E) Schematic diagram of the rheometer used for mechanical testing. F–H) Rheological analysis of HM vs HM‐TDN@AA‐HM: storage/loss modulus (F), viscosity (G), and tanδ (H). I) In vitro degradation in PBS over 16 days. J) Swelling ratio over time in PBS. K) Cumulative AA loading efficiency curve and L) release kinetics of AA from HM‐TDN@AA‐HM hydrogel (*n* = 3/group).

To develop an injectable scaffold capable of sustained AA release and mechanical adaptability for bone tissue engineering, we synthesized a photo‐crosslinkable hydrogel based on HAMA, and subsequently incorporated TDN@AA nanostructures to fabricate a composite system (HM‐TDN@AA). As shown in **Figure** **2**A, the resulting hydrogel precursor solution exhibited favorable injectability and underwent rapid gelation under 365 nm UV irradiation. Successful methacrylation of HA was confirmed by ^1^H NMR spectroscopy, as evidenced by the presence of characteristic peaks at 5.6–6.2 ppm corresponding to vinyl protons (Figure 2B). In addition, FTIR spectra of HM‐TDN@AA revealed prominent absorption peaks at 1314  and 1468 cm^−1^ (C─H bending of methacrylate groups), and 3400 cm^−1^ (O─H stretching), confirming the presence of methacryloyl and hydroxyl groups (Figure 2C). Notably, compared to HM–TDN, the HM‐TDN@AA group exhibited additional spectral features at 948 and 2855 cm^−1^, attributed to AA incorporation and molecular interactions within the hydrogel matrix.

The microstructure of the hydrogels was examined using SEM (Figure 2D). All groups displayed interconnected porous architectures, with the HM‐TDN@AA group exhibiting uniform and well‐organized pores, favorable for nutrient transport and cell infiltration. To characterize the viscoelastic behavior, a rheometer was used (Figure 2E), and rheological analysis revealed that both the storage modulus (G′) and loss modulus (G″) of HM‐TDN@AA were higher than HM alone (Figure 2F), indicating enhanced mechanical stiffness. Furthermore, HM‐TDN@AA displayed reduced viscosity (Figure 2G) and lower tanδ values (Figure 2H), suggesting improved shear‐thinning behavior and elasticity, which are beneficial for injectability and 3D printing compatibility.

The degradation kinetics of the hydrogels in PBS were monitored for 16 days (Figure 2I). In HM‐TDN@AA‐group, the HM‐TDN@AA incorporated did not significantly alter the degradation rate compared to HM. Swelling ratio analysis revealed that HM‐TDN@AA‐HM achieved rapid water absorption within 4 h and stabilized after 24 h, indicating excellent hydrogel hydration properties (Figure 2J). To evaluate the drug delivery performance of the hydrogel, the loading efficiency and release kinetics of AA were quantified. As shown in Figure 2K, the encapsulation efficiency of AA decreased with increasing initial concentrations, likely due to saturation of the DNA binding sites. Meanwhile, cumulative release analysis confirmed a sustained release profile, with ≈70% of AA released from the HM‐TDN@AA hydrogel over a 24 h period (Figure 2L).

Together, these results demonstrate that HM‐TDN@AA hydrogels possess favorable structural integrity, mechanical stability, and sustained drug release performance, establishing them as a promising platform for localized delivery in regenerative medicine.

### 2.3 HM‐TDN@AA Enhances Angiogenic Activity of Endothelial Cells In Vitro

**Figure 3:**
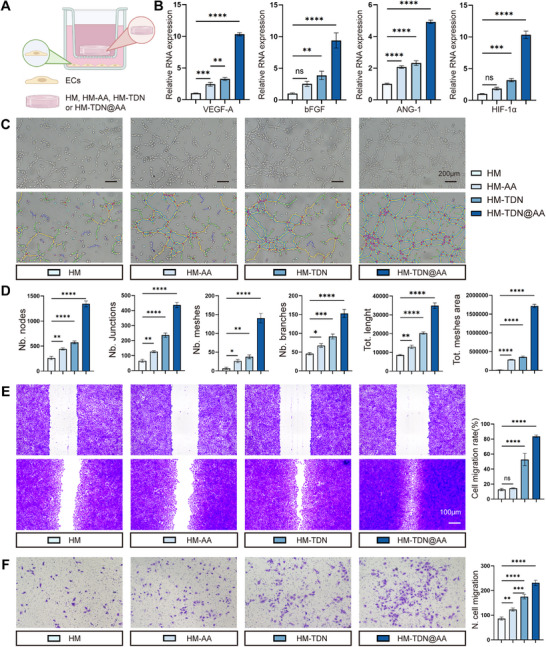
HM‐TDN@AA promotes endothelial angiogenic activity. A) Schematic diagram of the Transwell co‐culture system used to evaluate the angiogenic effects of HM‐TDN@AA on HUVECs. B) qPCR analysis of angiogenesis‐related genes (*VEGF‐A*, *bFGF*, *Ang1*, and *HIF1α*) in HUVECs after co‐culture with different treatment groups. C) Representative images of tube formation by HUVECs under different treatments. D) Quantitative analysis of tube formation metrics, including number of nodes, junctions, meshes, branches, total branch length, and mesh area. E) Representative images and quantification of the wound healing assay showing endothelial migration rates. F) Transwell migration assay showing the number of migrated HUVECs under different treatments. Data are presented as mean ± SD. ^*^ *p* < 0.05, ^**^ *p* < 0.01, ^***^ *p* < 0.001, ^****^ *p* < 0.0001; *n* = 3 in each independent experiments.

Given the pivotal role of neovascularization in supporting bone regeneration, we next explored the pro‐angiogenic potential of HM‐TDN@AA using human umbilical vein endothelial cells (HUVECs). CCK‐8 assay revealed that HM‐TDN@AA(20 µm) yielded the most pronounced proliferative effect on ECs, supporting its selection as the optimal working concentration (Figure S1A, Supporting Information). A Transwell co‐culture system was established, with HM‐TDN@AA placed in the upper chamber and HUVECs seeded in the lower chamber (**Figure** **3**A). Quantitative PCR revealed that HM‐TDN@AA significantly upregulated canonical pro‐angiogenic genes in HUVECs, including *VEGF‐A*, *bFGF*, *Ang1*, and *HIF1α* (Figure 3B). These factors are essential for endothelial cell proliferation, sprouting, and adaptation to hypoxic environments, suggesting that HM‐TDN@AA may modulate vascularization by transcriptionally reprogramming endothelial cells.

To functionally validate these transcriptional changes, we performed tube formation, wound healing, and Transwell migration assays. Notably, HM‐TDN@AA significantly enhanced the formation of tubular networks, as evidenced by increased numbers of nodes, junctions, meshes, branches, total branch length, and mesh area (Figure 3C,D). This suggests that the TDN‐based platform not only preserves but augments the angiogenic bioactivity of AA, likely by facilitating its uptake and retention in endothelial cells.

Consistently, HM‐TDN@AA‐treated HUVECs demonstrated accelerated scratch closure and increased migration rates, indicating enhanced motility and further reinforcing its angiogenic efficacy (Figure 3E,F). These findings collectively demonstrate that HM‐TDN@AA robustly enhances multiple facets of endothelial behavior required for angiogenesis. It is well established that AA exerts dose‐dependent effects on angiogenesis, functioning as anti‐angiogenic at higher concentrations (e.g., 40 µm) in tumor models,^[^ ^32^ ^]^ yet pro‐angiogenic at lower doses in regenerative contexts.^[^ ^14^, ^33^ ^]^ Our findings demonstrate that nano‐encapsulation of AA (20 µm) into a TDN (250 nm) scaffold markedly enhances its pro‐angiogenic efficacy by improving cellular uptake and bioavailability, offering a promising strategy to coordinate vascularization and osteogenesis during bone regeneration.

Enhanced angiogenesis during the early stages of bone healing is essential for establishing a regenerative microenvironment by ensuring adequate oxygen/nutrient supply, facilitating immune cell infiltration, and supporting subsequent bone matrix formation.^[^ ^2^, ^4^, ^34^ ^]^ Given that vascular invasion is a prerequisite for successful osteogenesis, the ability of HM‐TDN@AA to promote endothelial activation underscores its promise in creating a vascular‐supportive niche for coupled bone regeneration.

### 2.4 HM‐TDN@AA Inhibits Osteoclastogenesis and Promotes Osteogenesis In Vitro

**Figure 4:**
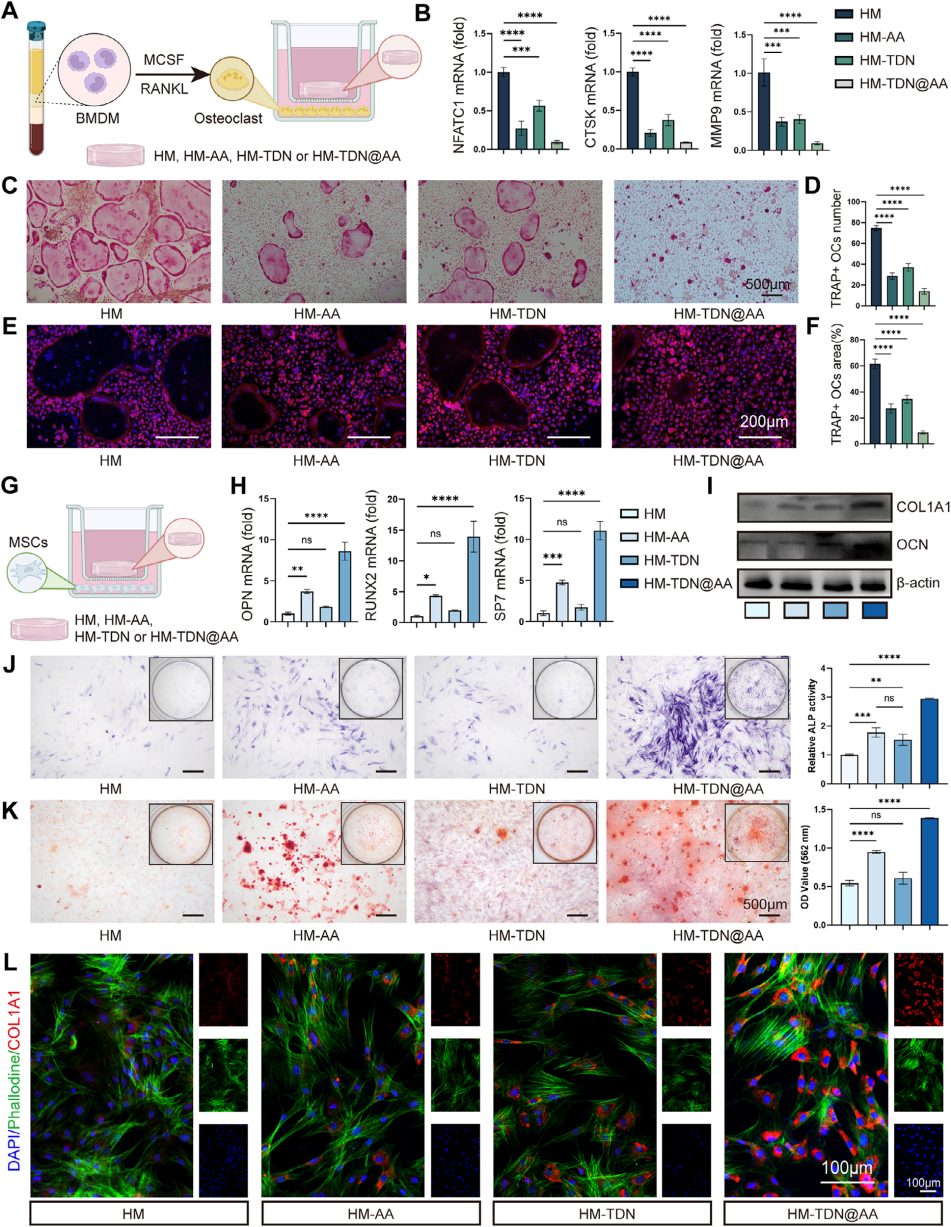
HM‐TDN@AA inhibits osteoclastogenesis and promotes osteogenesis in vitro. A) Schematic diagram illustrating the differentiation of BMDMs into osteoclasts under M‐CSF and RANKL stimulation and treatment with HM, HM‐AA, HM‐TDN, or HM‐TDN@AA. B) qRT‐PCR analysis of osteoclast‐related gene expression (NFATC1, CTSK, and MMP9) after treatment. C) TRAP staining of multinucleated osteoclasts. D–F) Quantification of TRAP‐positive cell number and area. E) IF staining showing actin ring formation in osteoclasts. G) Schematic of BMSCs undergoing osteogenic differentiation upon treatment. H,I) qRT‐PCR results showing mRNA levels of osteogenic markers (OPN, RUNX2, and SP7) and Western blot analysis of OCN and COL1A1 expression. J) Alizarin Red S staining and quantification of calcium nodule formation. K) ALP staining and activity assay. L) IF staining of COL1A1 (red), phalloidin (green), and nuclei (DAPI, blue) showing osteogenic morphology and matrix formation. Scale bars: 100 µm. ^ns^ *p* > 0.05, ^*^ *p* < 0.05, ^**^ *p* < 0.01, ^***^ *p* < 0.001; *n* = 3 per group, experiments were repeated in triplicate.

To explore how HM‐TDN@AA modulates bone remodeling, we initially investigated its impact on osteoclast lineage commitment. Bone marrow‐derived macrophages (BMDMs) were induced toward osteoclasts via M‐CSF and RANKL stimulation, subsequently exposed to HM, HM‐AA, HM‐TDN, or HM‐TDN@AA (**Figure** **4**A). Quantitative PCR revealed that HM‐TDN@AA significantly downregulated osteoclast‐specific markers, including *NFATC1*, *CTSK*, and *MMP9*, compared to control and single‐treatment groups (Figure 4B). Furthermore, TRAP staining indicated a significant decline in both the quantity and size of TRAP‐positive multinucleated osteoclasts within the HM‐TDN@AA treated group (Figure 4C,D), indicating a potent anti‐resorptive effect. Immunofluorescence (IF) staining (Figure 4E,F) further confirmed the suppression of osteoclast formation under HM‐TDN@AA treatment. The enhanced inhibitory effect of HM‐TDN@AA on osteoclast formation likely results from the synergistic actions of both components, as AA has been reported inhibit osteoclastogenesis,^[^ ^36^, ^37^, ^38^ ^]^ while TDNs improve intracellular delivery efficiency and bioavailability. This finding suggests that HM‐TDN@AA not only attenuates osteoclastic bone resorption but also modulates the inflammatory milieu during early bone healing.

Subsequently, we investigated the osteogenic potential of HM‐TDN@AA in bone marrow stromal cells (BMSCs) (Figure 4G). Gene expression profiling showed that HM‐TDN@AA significantly upregulated key osteogenic markers such as *OPN*, *RUNX2*, and *SP7* (Figure 4H). Western blotting revealed enhanced expression of osteocalcin (OCN) and type I collagen (COL1A1), confirming the pro‐osteogenic effect of the composite (Figure 4I). Functional mineralization was assessed by ALP and ARS staining, both of which showed a significant increase in calcium deposition and ALP activity in the HM‐TDN@AA group compared to controls (Figure 4J,K). Moreover, IF staining of COL1A1 and cytoskeletal organization demonstrated robust osteogenic differentiation under HM‐TDN@AA stimulation (Figure 4L).

Together, these results suggest that HM‐TDN@AA not only suppresses osteoclastogenesis but also enhances osteogenesis, possibly through a coordinated modulation of the osteoimmune environment. These dual effects are particularly beneficial in bone regeneration where a balance between bone resorption and formation is critical.^[^ ^8^ ^]^

### 2.5 HM‐TDN@AA Reshapes Early Angiogenesis and Modulates the Immune Microenvironment In Vivo

**Figure 5:**
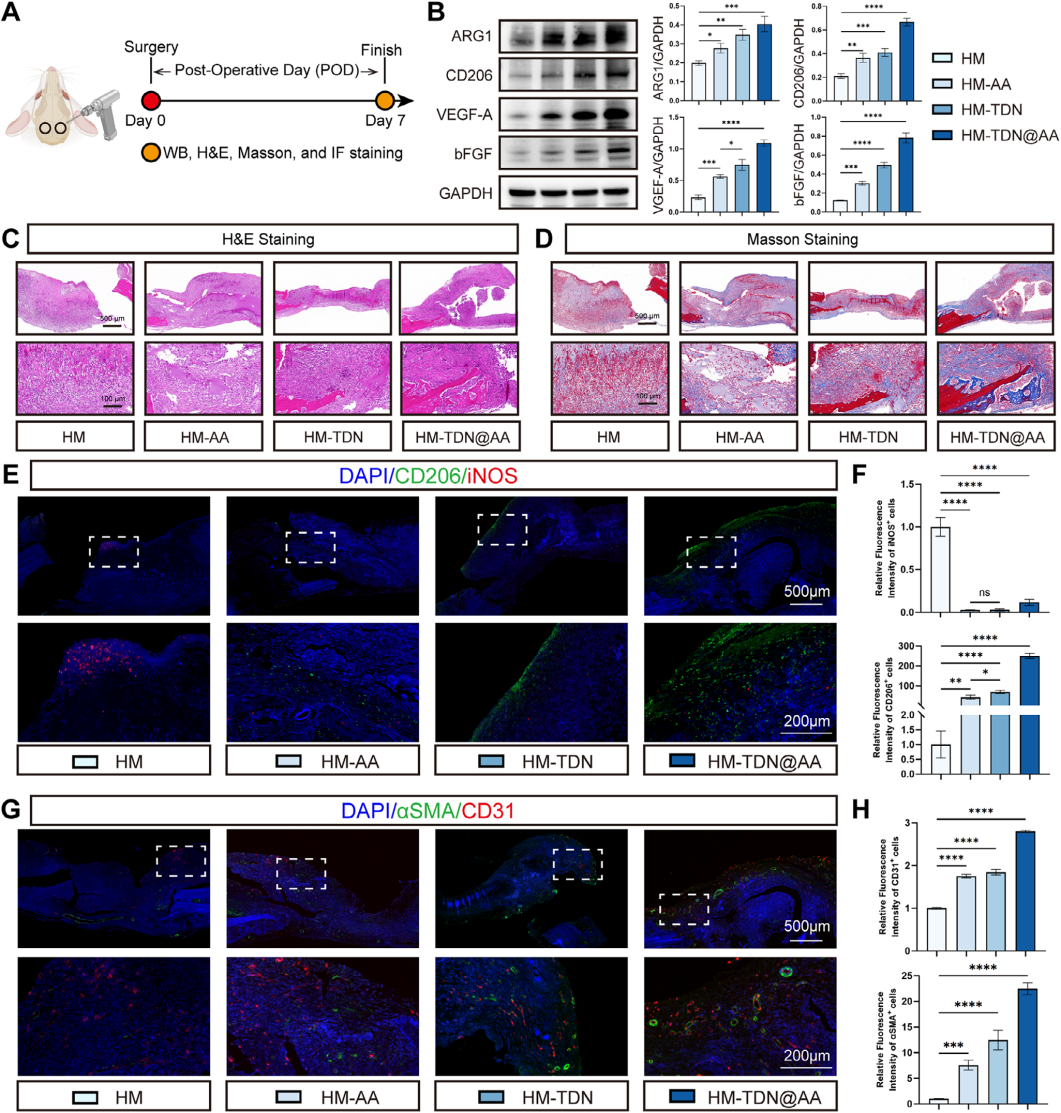
HM‐TDN@AA promotes early immunomodulation and angiogenesis in vivo. A) Schematic of the mouse calvarial defect model and experimental timeline. Angiogenesis‐related assessments were performed at postoperative day (POD) 7. B) Western blot analysis of ARG1, CD206, VEGF‐A, and bFGF expression in defect tissues at POD7, showing enhanced M2 macrophage polarization and pro‑angiogenic signaling in the HM‐TDN@AA group. C,D) Representative images of H&E staining (C) and Masson staining (D) at POD7. HM‐TDN@AA‐treated defects exhibited denser granulation tissue and greater collagen deposition, indicating enhanced early tissue organization. E) IF staining for iNOS (red) and CD206 (green) at POD7 to evaluate macrophage polarization. Insets show magnified regions of interest. F) Quantification of iNOS and CD206 fluorescence intensities, demonstrating a significant increase in M2 macrophage markers and a decrease in M1 markers in the HM‐TDN@AA group. G) IF staining for CD31 (red) and α‐SMA (green) showing blood vessel formation and maturation at POD7. DAPI labels nuclei (blue). H) Quantification of CD31^+^ and α‐SMA^+^ fluorescence signals, indicating improved angiogenic response in the HM‐TDN@AA group. Data are presented as mean ± SD. Statistical significance was assessed by one‐way ANOVA with Tukey's post hoc test: ^ns^ *p* > 0.05, ^*^ *p* < 0.05, ^**^ *p* < 0.01, ^***^ *p* < 0.001; *n* = 3 in each independent experiments.

To evaluate the in vivo effects of HM‐TDN@AA on the early bone healing environment,^[^ ^8^ ^]^ we established a critical‐sized calvarial defect model in mice and performed tissue harvesting on postoperative day (POD) 7 (**Figure** **5**A). H&E and Masson staining revealed that HM‐TDN@AA treatment promoted denser granulation tissue and enhanced collagen deposition, indicative of accelerated matrix remodeling at this early stage (Figure 5C,D). To quantitatively assess early immune and angiogenic responses, WB analyses were performed for polarization and angiogenesis‐related proteins. HM‐TDN@AA significantly upregulated the expression of ARG1 and CD206, indicating enhanced M2 macrophage polarization, and simultaneously elevated VEGF‐A and bFGF, which are key regulators of endothelial activation and angiogenesis (Figure 5B). These biochemical results provide direct evidence that HM‐TDN@AA modulates both immune polarization and angiogenic signaling at the protein level.

Consistently, IF staining for iNOS (M1‐like) and CD206 (M2‐like) revealed a distinct immunomodulatory effect of HM‐TDN@AA. Compared to the ctrl and AA groups, the HM‐TDN@AA group showed significantly reduced iNOS‐positive cells and a marked increase in CD206 expression (Figure 5E,F), indicating a polarization shift toward the anti‐inflammatory M2 phenotype. This shift is critical, as excessive M1 macrophage activation can prolong inflammation and impair healing, while M2 macrophages contribute to tissue repair by secreting pro‐regenerative cytokines and resolving inflammation.^[^ ^39^, ^40^ ^]^ These results suggest that HM‐TDN@AA fosters a regenerative immune microenvironment in the early stages of bone healing. Meanwhile, angiogenesis was assessed by immunostaining for CD31 and α‐SMA,^[^ ^41^ ^]^ which label endothelial cells and perivascular smooth muscle cells, respectively. The HM‐TDN@AA group demonstrated significantly higher CD31^+^ microvessel density and α‐SMA^+^ vessel maturity compared to other groups (Figure 5G,H), indicating robust neovascularization and vascular stabilization. This dual improvement in quantity and quality of vasculature highlights the pro‐angiogenic capacity of HM‐TDN@AA. The coordinated immunomodulation and vascular support are essential prerequisites for subsequent osteogenesis, as blood vessels deliver oxygen, nutrients, and osteoprogenitor cells into the defect area.^[^ ^40^, ^41^, ^42^ ^]^

Collectively, these results demonstrate that HM‐TDN@AA not only modulates immune responses to favor anti‐inflammatory macrophage polarization but also promotes early‐stage vascularization, thus synergistically establishing a pro‐regenerative niche that is critical for effective bone healing.

### 2.6 HM‐TDN@AA Enhances Late‐Stage Bone Regeneration and Matrix Remodeling in Calvarial Defects

**Figure 6:**
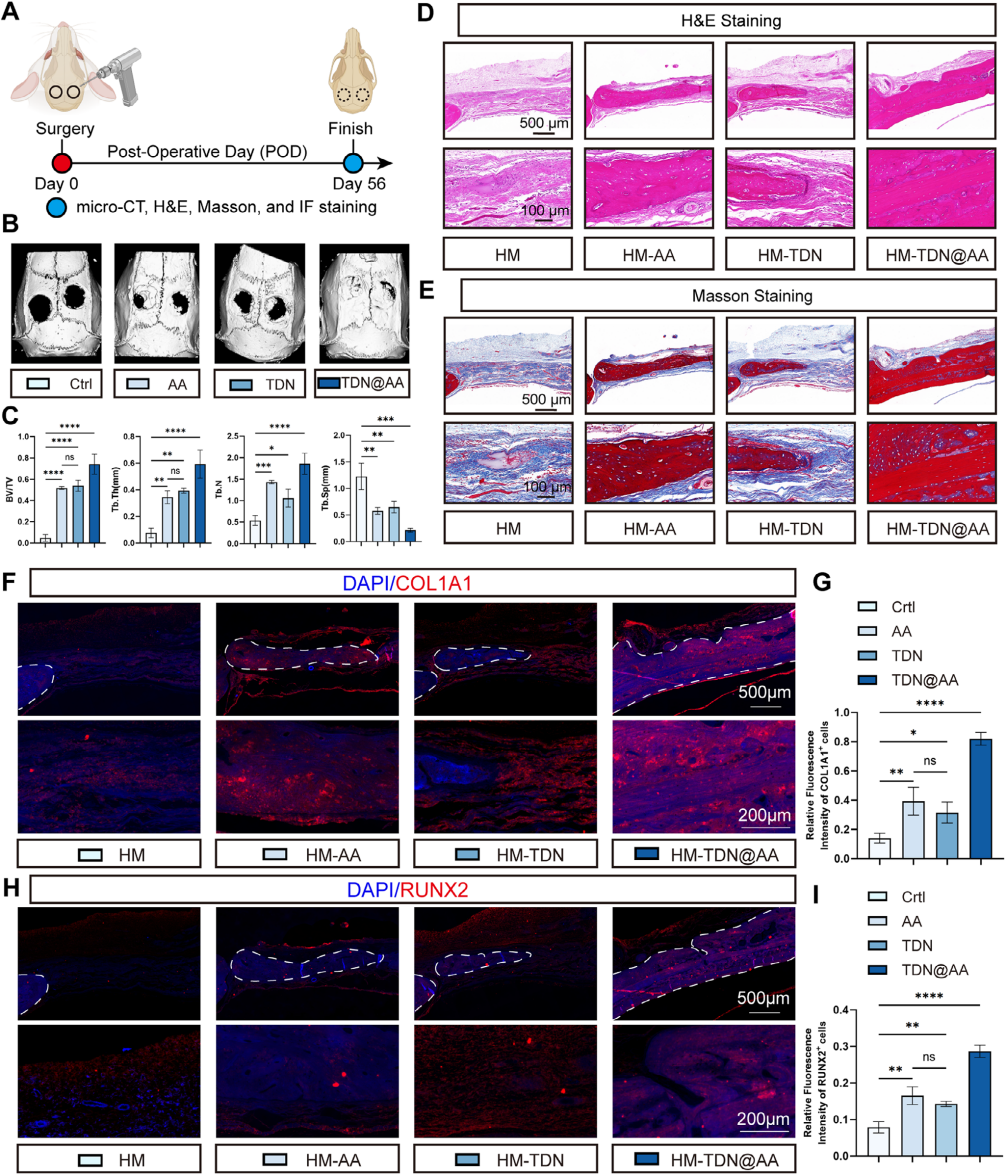
HM‐TDN@AA promotes bone regeneration at 8 weeks post‐surgery. A) Schematic of experimental timeline for late‐stage bone regeneration assessment. On postoperative day (POD) 56, micro‐CT, H&E staining, Masson's trichrome staining, and IF were performed to evaluate osteogenesis. B) Representative 3D reconstructed micro‐CT images of calvarial defects showing the extent of new bone formation in each group. C) Quantitative analysis of micro‐CT data including bone volume fraction (BV/TV), trabecular thickness (Tb.Th), trabecular number (Tb.N), and trabecular separation (Tb.Sp). HM‐TDN@AA treatment significantly increased BV/TV and Tb.Th, and decreased Tb.Sp compared to control and other groups. D,E) H&E (D) and Masson (E) staining of decalcified calvarial sections at POD56. HM‐TDN@AA‐treated samples showed denser and more organized collagenous matrix and new bone tissue. F) IF staining for COL1A1 (red) and DAPI (blue) to assess collagen matrix deposition in regenerated bone. G) Quantification of COL1A1 fluorescence intensity, indicating significantly higher collagen production in the HM‐TDN@AA group. H) IF staining for RUNX2 (red) and DAPI (blue), a key transcription factor for osteoblast differentiation. I) Quantification of RUNX2 fluorescence, showing that HM‐TDN@AA treatment resulted in markedly elevated osteogenic marker expression. Data are presented as mean ± SD. One‐way ANOVA followed by Tukey's test was used for statistical comparison. Significance levels: ^ns^ *p* > 0.05, ^*^ *p* < 0.05, ^**^ *p* < 0.01, ^***^ *p* < 0.001, ^****^ *p* < 0.0001; *n* = 3 rats in each group.

To evaluate the long‐term regenerative potential of HM‐TDN@AA in vivo, we examined bone healing at 8 weeks post‐surgery (POD56) (**Figure** **6**A). Unlike early time points which reflect inflammatory and vascular dynamics, the POD56 endpoint allows direct visualization of mature bone formation, tissue remodeling, and the net balance between osteogenesis and osteoclast activity. Micro‐CT imaging revealed substantial new bone formation in the HM‐TDN@AA group, in contrast to limited repair observed in the Ctrl, AA, and TDN groups (Figure 6B). Quantitative analysis confirmed that HM‐TDN@AA treatment resulted in significantly increased bone volume fraction (BV/TV), trabecular thickness (Tb.Th), and trabecular number (Tb.N), as well as decreased trabecular separation (Tb.Sp), indicating more complete and organized bone regeneration (Figure 6C). These findings demonstrate that HM‐TDN@AA promotes robust structural restoration of critical‐size defects. Histological staining further corroborated the micro‐CT results. Histological staining (H&E and Masson) of calvarial specimens revealed that extensive and mature new bone formation in the HM‐TDN@AA group, characterized by thicker trabeculae and dense collagen deposition compared to other groups (Figure 6D,E). These observations suggest that HM‐TDN@AA not only accelerates bone filling but also facilitates matrix remodeling and mineralization. To explore the underlying molecular changes, IF staining for COL1A1 and RUNX2 was performed. COL1A1, the major structural component of the bone extracellular matrix, was markedly upregulated in the HM‐TDN@AA‐treated group, as evidenced by stronger fluorescence intensity (Figure 6F,G). RUNX2, a master transcription factor for osteoblast differentiation, also showed significantly higher expression in the HM‐TDN@AA group (Figure 6H,I). These data support the notion that HM‐TDN@AA not only promotes osteoconduction but also enhances osteoinduction by activating osteogenic gene expression.

The superior performance of HM‐TDN@AA at this late healing stage can be attributed to its comprehensive modulation of the regenerative microenvironment throughout the bone repair process. Early in the healing phase, HM‐TDN@AA promotes M2 macrophage polarization and enhances neovascularization, both of which help establish an immune‐permissive and metabolically supportive niche for osteogenesis.^[^ ^43^, ^44^, ^45^ ^]^ At the same time, the inhibition of osteoclast differentiation, observed in vitro and confirmed at early in vivo time points, likely contributes to the preservation of newly formed bone matrix during remodeling. This combination of early immune and vascular regulation with sustained osteogenic support offers a distinct advantage over conventional therapies that often focus on a single pathway.^[^ ^41^, ^46^ ^]^ By coordinating multiple biological processes in a temporally organized manner, HM‐TDN@AA not only accelerates bone regeneration but also improves the quality and durability of the newly formed tissue.

### 2.7 Network Pharmacology and Molecular Dynamics Identify STAT3 as a Mechanistically Relevant Target of Asiatic Acid

**Figure 7:**
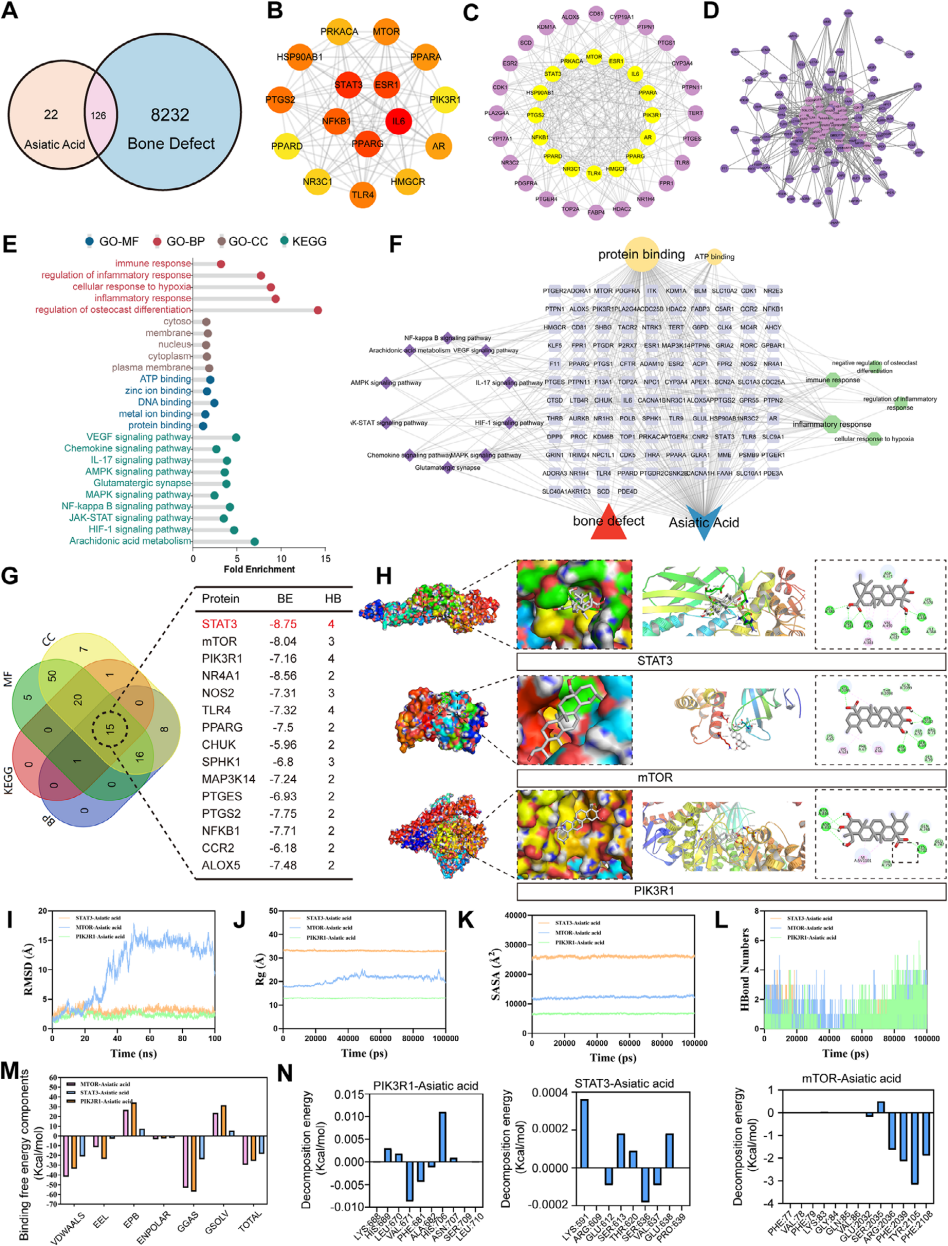
Network pharmacology and molecular docking reveal STAT3 as a key target of Asiatic acid in bone regeneration. A) Venn diagram illustrating the overlap between 22 predicted targets of Asiatic acid and 8232 bone defect‐related genes, yielding 126 shared targets. B,D) Protein‐protein interaction (PPI) network and identification of hub genes including STAT3, mTOR, and PIK3R1 using topological analysis. E,F) GO and KEGG enrichment analysis of overlapping targets, highlighting immune‐related and osteogenic pathways such as JAK‐STAT signaling, PI3K/Akt/mTOR axis, and VEGF signaling. G) Molecular docking results showing binding affinities of Asiatic acid to STAT3 (−8.75 kcal mol^−1^), mTOR (−8.04 kcal mol^−1^), and PIK3R1 (−7.16 kcal mol^−1^). H) Predicted binding modes and interaction residues in target proteins, including hydrophobic pockets and key signal‐transducing residues.

To elucidate the molecular basis by which HM‐TDN@AA regulates the regenerative microenvironment, we employed a systems pharmacology approach that combined drug‐target prediction with bone defect‐associated gene datasets. A total of 126 overlapping targets were identified from 22 predicted targets of Asiatic acid and 8232 bone regeneration‐related genes (**Figure** **7**A). PPI network analysis revealed that STAT3, mTOR, and PIK3R1 serve as central nodes in immunoregulatory, angiogenic, and osteogenic pathways (Figure 7B,D), suggesting that AA may exert pleiotropic effects across multiple cellular systems. GO and KEGG enrichment further linked these targets to pathways such as JAK‐STAT signaling, PI3K‐Akt‐mTOR, and VEGF signaling, which align with the observed biological responses of HM‐TDN@AA in modulating inflammation, vascularization, and bone regeneration (Figure 7E,F). These insights prompted us to explore whether AA directly interacts with these cores signaling proteins. We thus performed molecular docking and 100 ns molecular dynamics simulations of Asiatic acid with STAT3, mTOR, and PIK3R1. The docking results showed strong binding affinities, with calculated free energies of −8.75 kcal mol^−1^ (STAT3), −8.04 kcal mol^−1^ (mTOR), and −7.16 kcal mol^−1^ (PIK3R1), respectively (Figure 7G). Structural analyses revealed that AA engages these proteins via hydrophobic pockets and critical residues involved in signal transduction, including Tyr705 (STAT3) and Asp219 (PIK3R1) (Figure 7H). MD simulations confirmed the thermodynamic and conformational stability of the ligand‐target complexes. All three systems displayed low RMSD fluctuations, compact radius of gyration (Rg), and consistent hydrogen bonding patterns across 100 ns (Figure 7I–L). Energy decomposition revealed that binding was predominantly driven by van der Waals and electrostatic interactions, supporting the robustness of these interactions (Figure 7M,N).

While all three targets exhibited stable and energetically favorable interactions with AA, STAT3 was prioritized for further experimental validation due to its well‐established dual role in modulating both inflammation and bone regeneration.^[^ ^47^ ^]^ Notably, studies have reported that AA can both activate^[^ ^48^ ^]^ and inhibit^[^ ^49^ ^]^ STAT3 signaling, suggesting a concentration‐dependent regulatory mechanism. In addition, STAT3 serves as a central integrator of macrophage polarization, endothelial activation, and osteogenic differentiation,^[^ ^50^, ^51^, ^52^ ^]^ thereby positioning it as a key integrator of the immune‐vascular‐osteogenic axis and a promising mediator of the therapeutic effects exerted by the HM‐TDN@AA platform.

(I‐L) Molecular dynamics (MD) simulation trajectories for the three protein‐ligand complexes, including RMSD, radius of gyration (Rg), solvent‐accessible surface area (SASA), and hydrogen bond count across 100 ns. (M, N) Energy decomposition analyses of binding interactions, showing dominant contributions from van der Waals and electrostatic forces, with key residue interactions labeled.

### 2.8 HM‐TDN@AA Reprograms Mitochondrial Metabolism by Activating the STAT3 Signaling Axis

**Figure 8:**
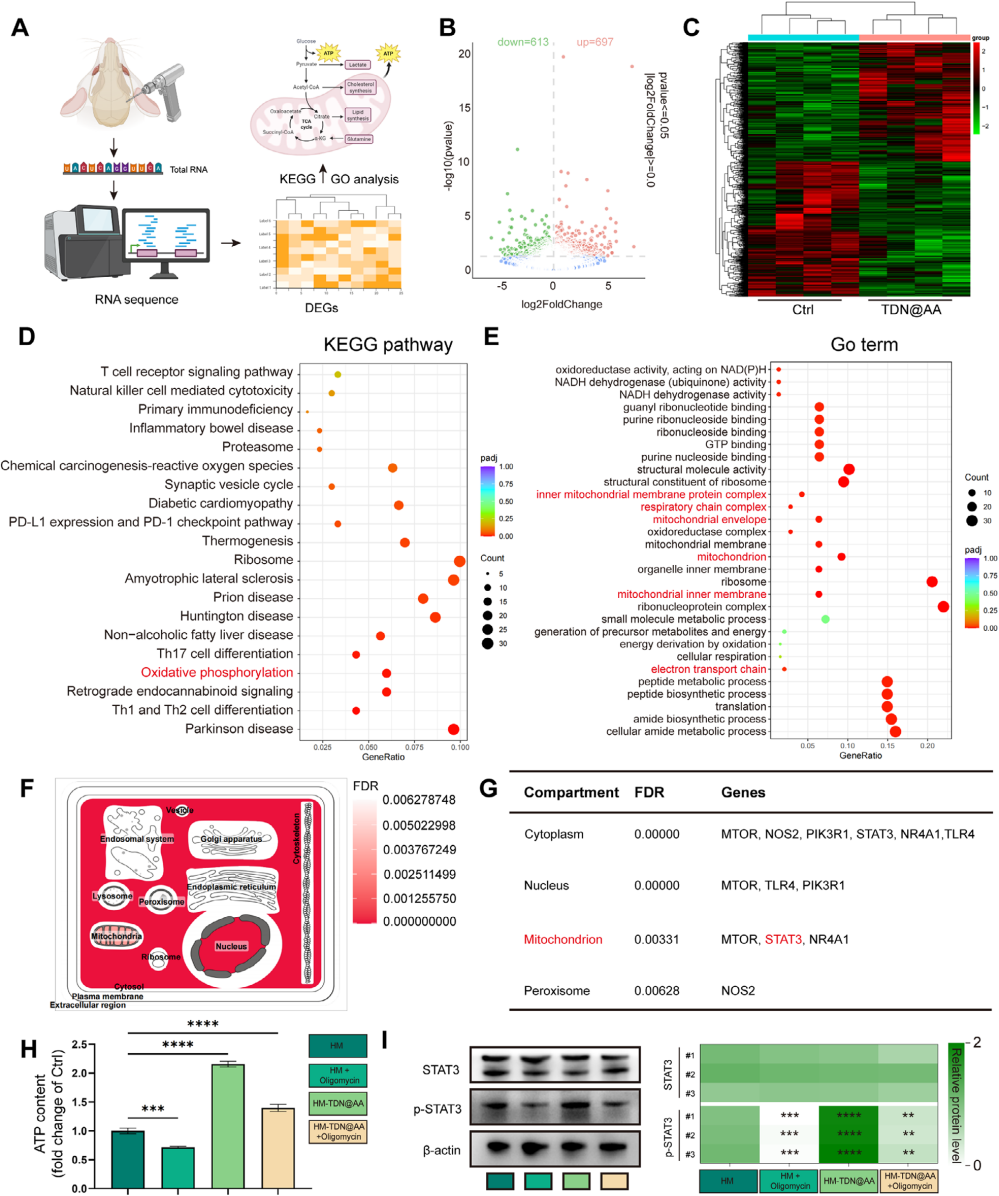
Transcriptomic analysis reveals mitochondrial metabolic reprogramming and immune pathway activation following HM‐TDN@AA treatment. A) Schematic of RNA‐seq on calvarial bone defects at POD7. B) Volcano plot showing differentially expressed genes (DEGs) between control and HM‐TDN@AA groups. C) Hierarchical clustering heatmap of DEGs. D) KEGG pathway analysis reveals enrichment of mitochondrial and oxidative phosphorylation‐related pathways. E) GO enrichment highlights mitochondrial ATP synthesis and respiratory complex assembly. F) Subcellular localization enrichment map, highlighting the mitochondrial compartment. G) Enrichment of DEGs in subcellular compartments; key DEGs localized in mitochondria include MTOR, STAT3, and NR4A1. H) ATP assay shows that HM‐TDN@AA significantly rescues ATP production impaired by oligomycin (^***^ *p* < 0.001, ^****^ *p* < 0.0001). I) Western blot and densitometric analysis demonstrate that HM‐TDN@AA reverses oligomycin‐induced downregulation of p‐STAT3. *n* = 3 in each group.

To gain mechanistic insight into how HM‐TDN@AA regulates the early regenerative microenvironment, we performed transcriptomic analysis of calvarial bone defects on POD7 (**Figure** **8**A). RNA sequencing identified 1310 differentially expressed genes (DEGs), among which genes involved in mitochondrial metabolism, ATP production, and immunoregulation were significantly enriched (Figure 8B,C). KEGG enrichment analysis showed that HM‐TDN@AA significantly activated mitochondrial‐related pathways, including oxidative phosphorylation, electron transport, and the Hippo and IL‐17 signaling pathways (Figure 8D). GO enrichment further supported this observation, with significant enrichment in processes related to mitochondrial metabolism, ATP biosynthesis, and respiratory chain complex assembly (Figure 8E). Importantly, these mitochondrial‐related annotations were consistent with earlier GO results from Asiatic acid's predicted targets (Figure 7), which also included ATP binding and oxidative regulation. This overlap supports the notion that mitochondrial metabolism may serve as a convergent point of AA‐mediated bioactivity. Emerging evidence highlights the pivotal role of mitochondria as central regulators in bone regeneration, orchestrating immune modulation, angiogenesis, and osteogenic‐osteoclastic balance through intercellular communication and metabolic signaling pathways.^[^ ^53^, ^54^ ^]^

Subcellular localization analysis^[^ ^55^ ^]^ showed that key genes upregulated by HM‐TDN@AA, including STAT3, mTOR, and NR4A1, were not only present in the cytoplasm and nucleus, but also enriched in mitochondria (Figure 8F,G). Among them, STAT3 has been widely implicated in regulating mitochondrial respiration and reactive oxygen species (ROS) generation, suggesting that its upregulation may mediate metabolic adaptation in the regenerating niche. Mitochondrial fitness is increasingly recognized as a central regulator of tissue regeneration.^[^ ^56^ ^]^ To confirm whether the pro‐osteogenic efficacy of HM‐TDN@AA is mediated through mitochondrial activation, we introduced oligomycin,^[^ ^53^ ^]^ an ATP synthase inhibitor, to functionally disrupt oxidative phosphorylation (OXPHOS). Oligomycin treatment led to a marked decrease in ATP levels, which was significantly restored by HM‐TDN@AA, consistent with its proposed role in mitochondrial activation (Figure 8H). Given the established role of STAT3 in mitochondrial transcription and bioenergetics, we evaluated its phosphorylation status. To determine the optimal working dose, we examined STAT3 phosphorylation in HUVECs exposed to increasing concentrations of HM‐TDN@AA. WB analysis revealed a clear bell‑shaped dose response, with maximal STAT3 activation observed at 20 µm, whereas higher concentrations (e.g., 80 µm) resulted in diminished phosphorylation (Figure S1B, Supporting Information). Accordingly, 20 µm, which had been used throughout our previous experiments, was further confirmed to be an appropriate working concentration, providing robust STAT3 activation while avoiding the reduced phosphorylation observed at higher doses. Furthermore, WB also confirmed that HM‐TDN@AA could reverse the suppression of p‐STAT3 caused by oligomycin, without altering total STAT3 expression (Figure 8I).

Together, these findings suggest that HM‐TDN@AA enhances mitochondrial bioenergetics, potentially via reactivation of the STAT3 signaling axis. This metabolic shift may act upstream to orchestrate immune regulation and angiogenesis, creating a regenerative microenvironment favorable for osteogenesis.

### 2.9 HM‐TDN@AA Activates Mitochondrial Metabolism in HUVECs and Promotes BMSC Osteogenesis via Paracrine Crosstalk

**Figure 9:**
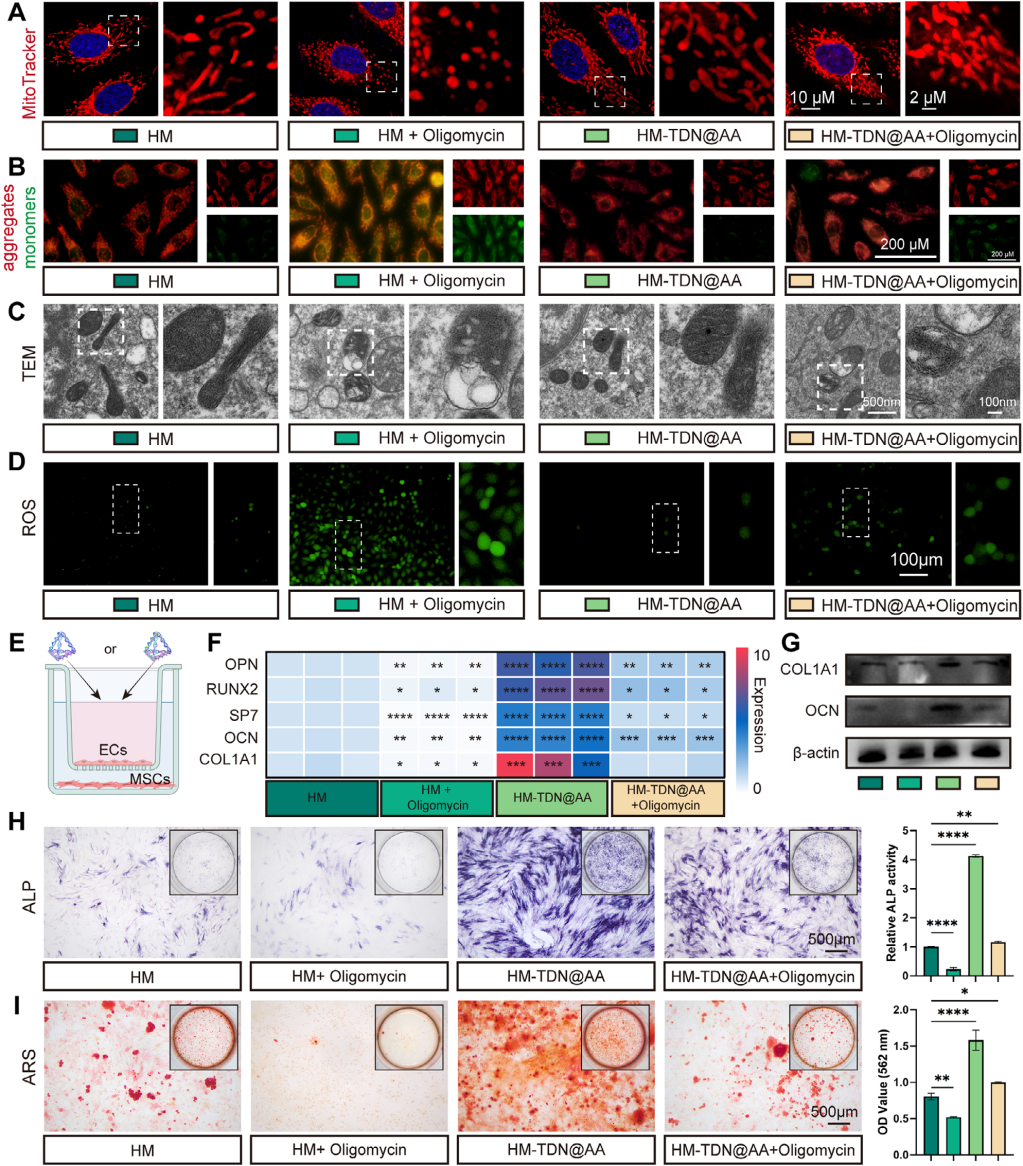
Mitochondria‐dependent metabolic activation is essential for the pro‐osteogenic effect of HM‐TDN@AA. A) MitoTracker staining of mitochondrial morphology in ECs treated with control, oligomycin, HM‐TDN@AA, or HM‐TDN@AA + oligomycin. HM‐TDN@AA restored tubular mitochondrial networks disrupted by oligomycin. B) JC‐1 staining showing mitochondrial membrane potential (red: aggregates, green: monomers). HM‐TDN@AA increased membrane potential, reversed by oligomycin. C) Transmission electron microscopy (TEM) of mitochondrial ultrastructure. HM‐TDN@AA enhanced cristae density and mitochondrial integrity. D) ROS detection using a fluorescent probe. HM‐TDN@AA reduced intracellular ROS levels, while oligomycin reversed this effect. E) Schematic of EC‐MSC co‐culture system for evaluating indirect osteogenic support. F) Heatmap of osteogenic gene expression (*OPN*, *RUNX2*, *SP7*, *OCN, COL1A1*) under different treatments. G) Western blot validation of OCN and COL1A1 expression. H) ALP staining and quantitative analysis. HM‐TDN@AA increased ALP activity, which was suppressed by oligomycin. I) Alizarin Red S staining and quantification of mineral deposition. HM‐TDN@AA enhanced matrix mineralization in a mitochondria‐dependent manner. Statistical significance: *^ns^p* > 0.05, ^*^p < 0.05, ^**^p < 0.01, ^***^p < 0.001; *n* = 3 in each independent experiments.

Given the enhancement of endothelial mitochondrial function observed in our earlier experiments, we next investigated whether structural and bioenergetic remodeling of mitochondria is central to the downstream regenerative outcomes. HM‐TDN@AA restored mitochondrial network integrity, as shown by elongated tubular morphology and dense MitoTracker signal, while oligomycin induced fragmentation and perinuclear clustering, typical of mitochondrial stress (**Figure** **9**A). Consistent with this, JC‐1 dye indicated enhanced mitochondrial membrane potential in the HM‐TDN@AA group, whereas oligomycin diminished polarization, confirming the bioenergetic dependence of HM‐TDN@AA's mitochondrial action (Figure 9B). TEM revealed that HM‐TDN@AA promoted denser cristae and healthier mitochondria ultrastructure, which is an essential prerequisite for sustained ATP output (Figure 9C). Functionally, HM‐TDN@AA decreased ROS production (Figure 9D), suggesting improved redox homeostasis. This is crucial as moderate ROS levels are needed for signaling, while excess impairs osteogenesis. The protective effect was lost with oligomycin, indicating that redox modulation by HM‐TDN@AA relies on functional mitochondria.

To further elucidate the effects of endothelial mitochondrial dysfunction on subsequent osteogenesis, we established an EC‐MSC co‐culture system to mimic the endothelial‐osteogenic interface (Figure 9E). qPCR and Western blot analysis revealed marked upregulation of RUNX2, SP7, OCN, and COL1A1 in the HM‐TDN@AA group, whereas oligomycin suppressed these markers (Figure 9F,G). Notably, HM‐TDN@AA restored ALP activity and calcium deposition even in the endothelial context, reinforcing that mitochondrial reprogramming enables intercellular osteogenic support (Figure 9H,I).

Together, these findings validate that HM‐TDN@AA exerts its regenerative effect through mitochondria‐centered metabolic activation, which orchestrates transcriptional and intercellular programs essential for angiogenesis and osteogenesis. The rescue of bone‐related markers in the presence of mitochondrial activation, and their loss upon inhibition, provide direct mechanistic evidence for mitochondria as the convergence point of HM‐TDN@AA action. This mitochondrial dependency underlines a paradigm where targeted metabolic enhancement at the vascular‐osteogenic interface can serve as a therapeutic entry point for complex tissue regeneration.

## 3 Conclusion

In this study, we developed a multifunctional HAMA‐based DNA nanostructure delivery system (HM‐TDN@AA) that integrates the angiogenic, immunomodulatory, and osteogenic activities of Asiatic acid (AA) with the programmable delivery capacity of TDNs and the injectable, mechanically adaptable properties of the HAMA hydrogel.Through a comprehensive series of in vitro and in vivo experiments, we demonstrated that HM‐TDN@AA significantly enhances vascularized bone regeneration by orchestrating multiple key regenerative processes. Mechanistically, HM‐TDN@AA promoted M2 macrophage polarization, suppressed osteoclast differentiation, and stimulated endothelial cell migration and tube formation, thereby establishing a pro‐healing immune‐vascular environment. In vivo RNA‐seq profiling further revealed that HM‐TDN@AA activates mitochondrial metabolic programs associated with energy production and biosynthesis. Functional validation using a mitochondrial inhibitor confirmed that metabolic reprogramming is a critical driver of the osteo‐inductive effect of HM‐TDN@AA. Importantly, network pharmacology and molecular docking identified STAT3 as a core regulatory node linking AA to immune‐metabolic signaling, suggesting a central role for STAT3‐mediated mitochondrial function in bone regeneration. Altogether, our findings not only shed light on the integrated mechanism of immune‐angiogenic and metabolic reprogramming by HM‐TDN@AA but also establish a rational framework for designing multifunctional bone biomaterials that target multiple regenerative checkpoints. This strategy holds great potential for clinical translation in large bone defect repair (Scheme 1).

## 4 Experimental Section

### Synthesis and Characterization of TDN and TDN@AA

Four single‐stranded DNA oligonucleotides (ssDNA1‐4; Table S1, Supporting Information) were synthesized and HPLC‐purified by Sangon Biotech (Shanghai, China). DNA tetrahedra (TDNs) were assembled by mixing equimolar amounts (1 µm) of each strand in TM buffer (10 mm Tris, 50 mm MgCl_2_, pH 8.0). The mixture was heated at 95 °C for 5 min and slowly cooled to 4 °C over 2 h to facilitate self‐assembly. To prepare TDN@AA, Asiatic acid (AA) was first dissolved in DMSO (10 mm) and then incubated with preassembled TDNs at a molar ratio of 40:1 (AA: TDN) for 6 h at room temperature to allow for noncovalent loading via hydrophobic and electrostatic interactions.

The structural integrity and mobility of TDN and TDN@AA were evaluated using 8% native polyacrylamide gel electrophoresis (PAGE). The absorbance profile of AA within the complex was assessed by UV–Vis spectroscopy, and drug loading efficiency was determined by comparing absorbance at 210 nm against a standard calibration curve.

Molecular docking was conducted using AutoDock Vina to simulate the interaction between AA and DNA grooves. The 3D structure of AA was obtained from the PubChem database, and DNA models were constructed based on canonical B‐form helices. Docking results were visualized using PyMOL and Discovery Studio to assess binding conformation and interaction types.

The hydrodynamic size and surface charge of TDN and TDN@AA were characterized using dynamic light scattering (DLS) and zeta potential measurements (Malvern Zetasizer Nano ZS). Morphological features were observed via transmission electron microscopy (TEM, JEOL JEM‐2100) following negative staining.

To evaluate internalization, the S1 strand was modified with Cy5 at the 5′ end. EPCs and JBMSCs were seeded in confocal dishes and incubated with Cy5‐labeled ssDNA, TDN, or TDN@AA for 24 h. After incubation, cells were washed with PBS, fixed with 4% paraformaldehyde, stained with DAPI for nuclei, and Phalloidin‐488 for the actin cytoskeleton. Intracellular fluorescence and cytoskeletal organization were observed using a confocal laser scanning microscope (LSM900, ZEISS Germany).

To assess lysosomal colocalization, HUVECs were seeded on glass‐bottom confocal dishes and allowed to adhere overnight. Cy5‐labeled TDN@AA was added to the culture medium at the designated working concentration and incubated with the cells for 6, 8, or 12 h. Following treatment, cells were washed three times with PBS and stained with LysoTracker Green (50 nm, Ex 488 nm) at 37 °C for 30 min to visualize lysosomes. Nuclei were counterstained with Hoechst 33342 (10 µg mL^−1^, Ex 405 nm) for 10 min. After a final PBS wash, images were acquired using a laser‐scanning confocal microscope to examine the intracellular distribution and lysosomal colocalization of Cy5‐TDN@AA.

### The Preparation of HM‐TDN@AA

To prepare the HA‐MA precursor, 10 g of hyaluronic acid (HA) was dissolved in 500 mL of deionized water under continuous mechanical stirring until a clear and homogeneous solution was obtained. Subsequently, 20 mL of methacrylic anhydride (MA) was added dropwise to the solution with vigorous stirring to ensure uniform dispersion. A 5 m NaOH solution (20 mL) was then introduced into the reaction mixture using a microinjection pump over 20–30 min to maintain the desired pH for methacrylation. The reaction was allowed to proceed overnight in an ice–water bath to suppress side reactions and maintain structural integrity.

After completion of the reaction, the solution was centrifuged at 7000 rpm for 15 min to remove insoluble residues. The supernatant was transferred into a dialysis bag (molecular weight cutoff: 8000 Da) and dialyzed against deionized water at room temperature for 3 days, with frequent water changes to ensure complete removal of unreacted reagents. The purified product was subsequently freeze‐dried to obtain HA‐MA as a white porous solid for further use.

For the preparation of HM‐TDN@AA hydrogel, lyophilized HA‐MA was reconstituted in PBS and thoroughly mixed with HM‐TDN@AA solution, which was preloaded with 250 nm DNA tetrahedrons and 20 µm Asiatic acid. The mixture was gently vortexed to ensure homogeneity and crosslinked under UV irradiation to form the final hydrogel construct for subsequent experiments.

To determine the encapsulation efficiency of AA within TDNs, the TDN@AA solution was subjected to centrifugal ultrafiltration, and the filtrate containing unencapsulated (free) AA was collected. The concentration of free AA was quantified by measuring its absorbance at 210 nm using a UV–Vis spectrophotometer and calculated based on a standard calibration curve.

For the drug release study, TDN@AA was dispersed in PBS (pH 7.4) and incubated at 37 °C under gentle shaking. At predetermined time intervals, samples were processed through ultrafiltration tubes to obtain the filtrate containing the released AA, which was quantified at 210 nm. An equal volume of fresh PBS was then added to the remaining samples to maintain constant volume and sustain sink conditions.

### Transwell Co‐Culture Setup

An indirect Transwell‐based co‐culture system was established to simulate paracrine signaling between hydrogel‐released HM‐TDN@AA and target cells. Specifically, pre‐formed HM‐TDN@AA hydrogel discs were placed in the insert (0.4 µm pore size, Corning), while HUVECs, MSCs, or osteoclast precursors (1 × 10⁵ cells/well) were seeded on the well surface.

### Quantitative Real‐Time PCR (qRT‐PCR)

Total RNA was isolated from HUVECs, BMSCs, and BMDM using TRIzol reagent (Invitrogen). cDNA was synthesized through reverse transcription using the RevertAid First Strand cDNA Synthesis Kit (Invitrogen, USA). qRT‐PCR was carried out with SYBR Green (Cat. A25742, Invitrogen, USA) on a StepOnePlus Real‐Time PCR system (Applied Biosystems). The transcription levels of genes involved in angiogenesis (*bFGF*, *VEGFA*, *Ang1*, and *HIF1α*), osteogenic markers (*RUNX2*, *OPN*, *SP7*, *OCN*, and *COL1A1*), and osteoclastogenesis‐associated genes (*NFATC1*, *CTSK*, and *MMP9*) were quantified using the 2^−ΔΔCt^ method, with GAPDH serving as the internal reference. All primers were synthesized by Sangon Biotech (Shanghai, China), and the full list is provided in Table S2 (Supporting Information).

### Tube Formation Assay

The angiogenic capacity of HUVECs was evaluated using a Matrigel‐based tube formation assay. Briefly, Matrigel (Corning, USA) was added to 24 well plate and incubated at 37 °C for 30 min to allow gelation. HUVECs were resuspended at a density of 1.5 × 10⁵ cells/mL and seeded into the wells. Subsequently, HM, HM‐AA, HM‐TDN, or HM‐TDN@AA was added in the upper chamber of Transwell inserts. After 6 h, the tube structures were visualized under an inverted fluorescence microscope. Tube formation was quantified by analyzing three random microscopic fields per sample using ImageJ software with the Angiogenesis Analyzer plugin.

### Transwell Assay

Transwell assays were performed to assess the migratory ability of HUVECs. Briefly, 8 × 10^3^ cells were suspended in 400 µL serum‐free DMEM and seeded into the upper chamber of Transwell inserts (8.0 µm pore size, Corning, USA) in 24‐well plates. The lower chambers were filled with 700 µL complete medium containing HM, HM‐AA, HM‐TDN, or HM‐TDN@AA. After 24 h of incubation at 37 °C, non‐migrated cells on the upper membrane surface were gently removed with a cotton swab. Migrated cells on the lower membrane were fixed with 4% paraformaldehyde (PFA) for 20 min, stained with 0.1% crystal violet (Solarbio, China) for 30 min, imaged under an inverted microscope, and analyzed using ImageJ software.

### Wound Healing Assay

For wound healing analysis, HUVECs were cultured in 12‐well plates until 80% confluence. After serum deprivation overnight, a straight scratch was made using a 200 µL pipette tip. Detached cells were washed away with PBS, and the remaining monolayer was cultured in serum‐free DMEM, and HM, HM‐AA, HM‐TDN, or HM‐TDN@AA was added in the upper chamber of Transwell inserts. Scratch areas were imaged at 0 and 24 h using a microscope (Leica DMi8) and calculated migration rate using ImageJ software.

### Osteoclastogenesis Assay and TRAP Staining

Osteoclast differentiation was induced by treating bone marrow‐derived macrophages (BMDMs) with 30 ng/mL M‐CSF and 50 ng mL^−1^ RANKL (R&D, USA) in the presence of various formulations (Ctrl, AA, TDN, and HM‐TDN@AA). Cells were cultured for 6 days with media changed every 2 days. TRAP staining was performed using a commercial TRAP kit (Cat. 294–67001, wako, Japan,). TRAP‐positive multinucleated cells (≥3 nuclei) were counted under a microscope, and osteoclast area was quantified using ImageJ.

### Alizarin Red S (ARS) and Alkaline Phosphatase (ALP) Staining

On day 7 of osteogenic induction, cells were fixed and stained using a BCIP/NBT ALP staining kit (Beyotime, Cat. C3206) to evaluate ALP activity, which was further quantified using an ALP assay kit (Beyotime, Cat. P0321S). After 14 days of induction, mineralized matrix deposition was assessed by Alizarin Red S staining (Solarbio) and quantified using 10% cetylpyridinium chloride.

### IF Staining of BMSCs or BMDM

BMSCs or BMDMs were fixed with 4% PFA for 15 min. For BMDMs, cytoskeletal staining was performed directly using Phalloidin (Alexa Fluor 594, Invitrogen). BMSCs were further permeabilized with 0.1% Triton X‐100 and blocked with 3% bovine serum albumin (BSA) for 1 h. Cells were then incubated overnight at 4 °C with a primary antibody against COL1A1 (1:200, Proteintech, 14695‐1‐ap), followed by Alexa Fluor‐conjugated secondary antibodies. The cytoskeleton was stained with Phalloidin (Alexa Fluor 488), and nuclei were counterstained with DAPI. Images were acquired using a laser scanning confocal microscope (LSM900, ZEISS Germany).

### Western Blot Analysis

Cells were lysed using RIPA buffer (Beyotime, China) supplemented with protease and phosphatase inhibitor cocktails (Thermo Scientific, USA). Lysates were centrifuged at 12,000 g for 15 min at 4 °C to collect supernatants. Protein concentrations were determined using a BCA Protein Assay Kit (Beyotime, China). Equal amounts of protein (20 µg) were separated by 10% SDS‐PAGE and transferred onto PVDF membranes (0.22 µm, Millipore, USA). Membranes were blocked with 5% non‐fat milk in TBST for 1 h at room temperature, and incubated overnight at 4 °C with the following primary antibodies: anti‐VEGFA(1:1000, 26157‐1‐AP, Proteintech), anti‐bFGF(1:1000, 11234‐1‐AP, Proteintech), anti‐Arg‐1(1:1000, #93 668, CST), anti‐CD206(1:1000, #24 595, CST), anti‐STAT3 (1:5000, Abcam, ab119352), anti‐p‐STAT3 (1:2000, Abcam, ab76315), anti‐OCN (1:1000, Proteintech, 23418‐1‐ap), and anti‐COL1A1 (1:1000, Proteintech, 14695‐1‐ap). Subsequent to primary antibody incubation, membranes were incubated with HRP‐conjugated goat anti‐rabbit secondary antibodies (1:2000, Beyotime, A0216) or goat anti‐mouse secondary antibodies (1:2000, Beyotime, A0208) for 1 h at room temperature. Protein bands were visualized using an ECL reagent (Millipore, P90719) and captured with a chemiluminescence imaging system (Bio‐Rad). β‐Actin (1:1000, Beyotime, AA128) was used as a loading control, and band intensities were quantified using ImageJ software.

### Animal Surgery

All animal experiments, including surgical interventions and postoperative antibiotic use, were conducted in compliance with protocols approved by the Animal Ethics Committee of Shanghai Tenth People's Hospital (Approval No. SHDSYY‐2024‐3258).

Male Sprague‐Dawley rats (8 weeks old, 250 g) were anesthetized using Zoletil 50 (60 µL/20 g; Virbac, France) in combination with dexmedetomidine hydrochloride (15 µL/20 g; Orion Corporation, Finland) to reduce procedural stress. Two symmetrical calvarial critical‐size bone defects (5 mm in diameter) were created using a trephine drill under aseptic conditions. Circular hydrogel implants (5 mm diameter × 1 mm height) containing HM, HM‐AA (20 µm), HM‐TDN, or HM‐TDN@AA were implanted in situ into the calvarial bone defects in rats (*n* = 5 per group). Animals were sacrificed at postoperative day (POD) 7 and POD56 for angiogenesis and osteogenesis evaluation, respectively.

### Histology and IF Analysis for In Vivo

At predetermined time points, calvarial bone samples were collected and fixed in 4% paraformaldehyde for 48 h, followed by decalcification in 10% EDTA (pH 7.4) at 4 °C for 2–3 weeks with regular solution changes. Samples were dehydrated, paraffin‐embedded, and sectioned at 5 µm thickness. Hematoxylin‐eosin (H&E) staining was performed to assess general tissue morphology. Briefly, paraffin sections were deparaffinized, rehydrated, and stained with hematoxylin for 5 min, followed by eosin for 2 min. Slides were dehydrated and sealed with neutral resin. Masson's trichrome staining was used to evaluate collagen deposition and matrix maturation. Sections were stained using a commercial Masson's trichrome staining kit (Solarbio, China). For IF staining, deparaffinized sections underwent antigen retrieval in citrate buffer (pH 6.0) at 95 °C for 20 min, followed by permeabilization in 0.1% Triton X‐100 for 15 min. Sections were blocked and incubated overnight at 4 °C with primary antibodies against CD31 (1:2000, Abcam, ab281583), aSMA (1:2000, Boster, bm0002), iNOS (1:200, Abcam, ab178945), CD206 (1:1000, CST, 24 595), RUNX2 (1:500, Proteintech, 20700‐1‐ap), or COL1A1 (1:200, Proteintech, 14695‐1‐ap). After PBS washes, appropriate Alexa Fluor‐conjugated secondary antibodies (Invitrogen) were applied for 1 h at room temperature. Nuclei were counterstained with DAPI (Solarbio), and sections were mounted using anti‐fade mounting medium. Fluorescence images were acquired using a Leica DMi8 microscope, and quantitative analysis of fluorescence intensity was performed using ImageJ software.

### Network Pharmacology Analysis

Putative targets of Asiatic acid were predicted from multiple databases including Pubchem, SwissTargetPrediction, and Super‐PRED. Bone defect‐related genes were retrieved from GeneCards and OMIM databases. The overlapping targets between drug and disease were visualized using Venn diagrams. Protein‐protein interaction (PPI) networks were constructed via the STRING database and visualized in Cytoscape software (v3.10.0). Enrichment analyses for Gene Ontology (GO) and KEGG pathways were conducted using the Metascape platform.

### Molecular Docking and Dynamics Simulation

The 3D structures of candidate target proteins were obtained from the Protein Data Bank (PDB), and the chemical structure of Asiatic acid (AA) was retrieved from PubChem and processed using ChemDraw. Molecular docking was performed with AutoDock 4.2.6, allowing ligand flexibility and rigid receptor conformation. Docking results were visualized using PyMOL and Discovery Studio, with binding energy and hydrogen bond number used as evaluation criteria. Molecular dynamics (MD) simulations were conducted using GROMACS 2022. Protein and ligand topologies were generated using the CHARMM36 and CGenFF force fields, respectively. Systems were solvated in TIP3P water boxes and neutralized with counterions. After energy minimization, 100 ns MD simulations were run under NPT conditions at 310 K. RMSD, Rg, SASA, H‐bonds, and MM‐PBSA binding free energy were analyzed to assess structural stability and binding affinity. Subcellular localization of DEGs was predicted using the SubcellulaRVis database.^[^ ^55^ ^]^

### RNA‐Sequencing and Bioinformatic Analysis

Calvarial bone defect tissues were harvested 7 days after surgery for RNA extraction and sent for high‐throughput RNA sequencing (Novogene, Beijing, China) using the Illumina NovaSeq 6000 platform. Quality control was performed using FastQC and low‐quality reads were filtered. Clean reads were aligned to the mouse reference genome (GRCm38) using HISAT2. Differentially expressed genes (DEGs) were identified with DESeq2 (|log2FoldChange| > 1, adjusted p‐value < 0.05). GO and KEGG enrichment analyses were performed using the ClusterProfiler R package. Heatmaps and volcano plots were generated using the ggplot2 and pheatmap packages.

### Quantification of Intracellular ATP Concentration

HUVECs (1 × 10⁵ cells per well) were seeded in 24‐well plates and treated with HM or HM‐TDN@AA for 24 h. In a subset of experiments, oligomycin (2 µm) was added 2 h before sample collection to inhibit ATP synthase. Cells were then lysed using the Enhanced ATP Assay Kit (S0027, Beyotime, China). Luminescence was recorded using GloMax Discover.

### MitoTracker Red Staining

HUVECs were incubated with 50 nm MitoTracker Red CMXRos (Invitrogen, M7512) at 37 °C for 15 min. Following PBS rinsing, nuclei were counterstained with Hoechst (Cat. C1027, Beyotime, China). Imaging was performed using a confocal laser scanning microscope (LSM900, ZEISS, Germany).

### JC‐1 Assay

The mitochondrial membrane potential was evaluated using the JC‐1 assay kit (Cat. C2003S, Beyotime, China). Following treatment, cells were stained with JC‐1 dye (5 µg mL^−1^) at 37 °C. Red fluorescence indicates JC‐1 aggregates (high membrane potential) and green indicates JC‐1 monomers (low membrane potential).

### Transmission Electron Microscopy (TEM)

Cells were initially fixed overnight at 4 °C using 2.5% glutaraldehyde, followed by post‐fixation with 1% osmium tetroxide. After ethanol gradient dehydration, the samples were embedded in epoxy resin, ultrathin‐sectioned, and stained with uranyl acetate and lead citrate. Observations were conducted using a TEM system (HT7800, CanScan, Hitachi, Tokyo, Japan).

### Intracellular ROS Detection

Reactive oxygen species were assessed using the ROS Detection Kit (Cat. S0033S, Beyotime, China). After treatment, cells were incubated with 10 µm L^−1^ DCFH‐DA at 37 °C for 20 min in the dark. Excess dye was removed by PBS, and fluorescence was captured.

### Statistical Analysis

All quantitative results were expressed as mean values with corresponding standard deviations (SD). Comparisons between two independent groups were conducted using an unpaired two‐sided Student's t‐test. For comparisons involving more than two groups, one‐way ANOVA followed by Tukey's post hoc test was applied. Statistical analyses and graphing were performed using GraphPad Prism 9.0. A p‐value less than 0.05 was considered to indicate statistical significance (*p* < 0.05, *p* < 0.01, *p* < 0.001, *p* < 0.0001).

## Conflict of Interest

The authors declare no conflict of interest.

## Author Contributions

Y. H, Y. Z., and Y. F contributed equally to this work. Y.H. and Y.F. supervised and conceived of the study. H.X. and B.G. designed and completed the experiments. Q.Y provided resources. C.H validated the data. Y.F. and Z.Y. collected and analyzed the data. P.Z. wrote the manuscript. K.L., Y.X., and Y.H. provided help during data collection and analysis.

## Supporting information



Supporting Figure 1

Supporting Table 1

Supporting Table 2

## Data Availability

The data that support the findings of this study are available from the corresponding author upon reasonable request.
