# Supplementary figures and images for: A DNA Tetrahedron Delivery Asiatic Acid to Reprogram Mitochondrial Metabolism for Promoting Bone Regeneration via STAT3 Phosphorylation
Source: Adv Sci (Weinh). 2025 Dec 19;13(10):e18796. doi: 10.1002/advs.202518796 (PMC12915104; doi:10.1002/advs.202518796)

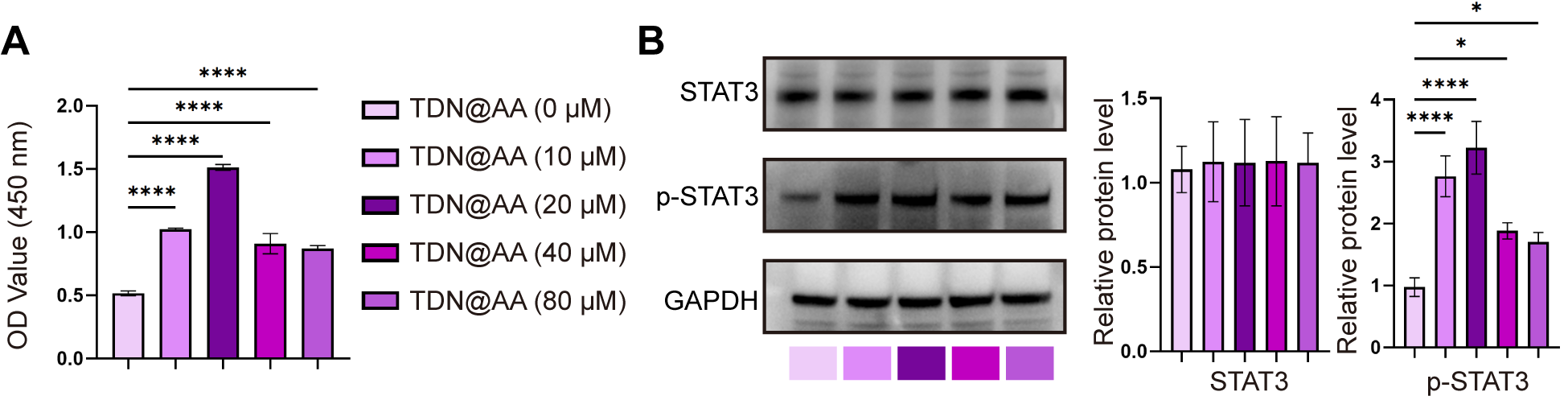

Supplement: Supplementary file 1 — Supporting Figure 1 [file ADVS-13-e18796-s003.tif]
