# Supplementary material for: A DNA Tetrahedron Delivery Asiatic Acid to Reprogram Mitochondrial Metabolism for Promoting Bone Regeneration via STAT3 Phosphorylation
Source: Adv Sci (Weinh). 2025 Dec 19;13(10):e18796. doi: 10.1002/advs.202518796 (PMC12915104; doi:10.1002/advs.202518796)
Supplement: Supplementary file 2 — Supporting Table 1 [file ADVS-13-e18796-s002.docx]

Table S1 Base sequence of single-stranded DNAs (ssDNAs) used to synthesize TDN.

| ssDNA | Sequences (from 5′ to 3′) |
| --- | --- |
| S1 | ATTTATCACCCGCCATAGTAGACGTATCACCAGGCAGTTGAGACGAACATTCCTAAGTCTGAA |
| S2 | ACATGCGAGGGTCCAATACCGACGATTACAGCTTGCTACACGATTCAGACTTAGGAATGTTCG |
| S3 | ACTACTATGGCGGGTGATAAAACGTGTAGCAAGCTGTAATCGACGGGAAGAGCATGCCCATCC |
| S4 | ACGGTATTGGACCCTCGCATGACTCAACTGCCTGGTGATACGAGGATGGGCATGCTCTTCCCG |
