# Supplementary material for: A DNA Tetrahedron Delivery Asiatic Acid to Reprogram Mitochondrial Metabolism for Promoting Bone Regeneration via STAT3 Phosphorylation
Source: Adv Sci (Weinh). 2025 Dec 19;13(10):e18796. doi: 10.1002/advs.202518796 (PMC12915104; doi:10.1002/advs.202518796)
Supplement: Supplementary file 3 — Supporting Table 2 [file ADVS-13-e18796-s001.docx]

Table S2-1 Primers sequences for qRT-PCR in BMSCs or BMDM.

| mRNA | Primer (from 5′ to 3′) | Sequence |
| --- | --- | --- |
| RUNX2 | Forward | GCACCCAGCCCATAATAGA |
|  | Reverse | TTGGAGCAAGGAGAACCC |
| SP7 | Forward | AGCTGCCTACTTACCCGTCTGA |
|  | Reverse | TGCCCACTATTGCCAACTGC |
| OCN | Forward | TGAGGACCCTCTCTCTGCTC |
|  | Reverse | GGGCTCCAAGTCCATTGTT |
| Col-1 | Forward | TGTTGGTCCTGCTGGCAAGAATG |
|  | Reverse | GTCACCTTGTTCGCCTGTCTCAC |
| OPN | Forward | CCAAGCGTGGAAACACACAGCC |
|  | Reverse | GGCTTTGGAACTCGCCTGACTG |
| GAPDH | Forward | CCTGCACCACCAACTGCTTA |
|  | Reverse | GGCCATCCACAGTCTTCTGAG |

Table S2-2 Primers sequences for qRT-PCR in HUVECs.

| mRNA | Primer (from 5′ to 3′) | Sequence |
| --- | --- | --- |
| VEGF | Forward | ATCGAGTACATCTTCAAGCCAT |
|  | Reverse | GTGAGGTTTGATCCGCATAATC |
| FGF | Forward | CATCAAGCTACAACTTCAAGCA |
|  | Reverse | CCGTAACACATTTAGAAGCCAG |
| Ang-1 | Forward |  |
|  | Reverse |  |
| HIF-1α | Forward | CTAACTAGCCGAGGAAGAACTATGAAC |
|  | Reverse | CACTGAGGTTGGTTACTGTTGGTATC |
| GAPDH | Forward | TGTTCGTCATGGGTGTGAAC |
|  | Reverse | ATGGCATGGACTGTGGTCAT |
